# Gender differences in the association between multimorbidity and depression in older Korean adults: an analysis of data from the National Survey of Older Koreans (2011-2017)

**DOI:** 10.4178/epih.e2022049

**Published:** 2022-05-24

**Authors:** SeoYeon Hwang, Jin Young Nam, Jeong Hyun Ahn, Soojin Park

**Affiliations:** Department of Healthcare Management, Eulji University, Sungnam, Korea

**Keywords:** Depression, Multimorbidity, Gender differences, Comorbidity

## Abstract

**OBJECTIVES:**

Previous studies have shown that people with multimorbidity have a higher risk of depression than those without multimorbidity. However, few studies have examined the association between depression and multimorbidity in men and women separately. Since the rates of depression and multimorbidity are different in men and women, it is necessary to examine whether gender differences affect their association.

**METHODS:**

This study included 30,138 participants (aged ≥ 65 years) from the National Survey of Older Koreans (2011-2017). Depression was defined using the Korean version of the Geriatric Depression Scale (SGDS-K). Multimorbidity was defined as people who had 2 or more chronic diseases, including arthritis, diabetes, heart disease, hypertension, pulmonary disease, cancer, stroke, or osteoporosis. Multiple logistic regression analysis was performed to determine the association between depression and multimorbidity.

**RESULTS:**

In total, 22.2% and 30.7% of men and women, respectively, had depression. Those with multimorbidity had a higher risk of depression than those without chronic conditions; specifically, the difference in risk among men was greater than that among women. Age was considered a moderator for women. While the effects of pulmonary disease, stroke, and cancer were especially substantial in the integrated analysis, gender differences were observed related to various chronic conditions comorbid with heart disease.

**CONCLUSIONS:**

There are gender differences in the association between multimorbidity and depression among older Korean adults. Therefore, gender-specific care should be provided to reduce depression in older adults with multimorbidity.

## INTRODUCTION

Depression in older adults is a common psychiatric disorder that diminishes their quality of life [1]. Depression should not be considered a typical side effect of aging, even if it occurs frequently. Depression tends to worsen with age [2] and can cause mortality by suicide or medical illnesses [3]. Factors known to be related to depression in older adults include gender (women in particular), chronic somatic illnesses, disability, poor social support, and bereavement [4-6].

Multimorbidity, which refers to the presence of 2 or more chronic diseases in an individual, is also associated with depression [7]. The presence of multimorbidity increases steadily with age [8,9]; accordingly, the number of patients with multimorbidity is growing rapidly as aging increases worldwide [10]. Moreover, multimorbidity elevates the risk of death, disability, poor quality of life, hospitalization, and instrumentalization [11,12]. For these reasons, the U.S. Department of Health and Human Services has called for researchers to focus on multimorbidity rather than on individual chronic conditions given the powerful effects of multimorbidity on individuals’ lives [13].

The association between depression and multimorbidity has been examined in many studies. A meta-analysis of 40 studies found that people with multimorbidity had a 2 times higher risk of depression than those with 1 chronic disease and approximately a 3 times higher risk of depression than those without any chronic diseases [14]. However, a recent longitudinal study of 2,002 community-dwelling older people from Canada, Brazil, Colombia, and Albania found that multimorbidity was not a risk factor for depression regardless of cultural differences [15]. In response to these conflicting results, this study examined the association between depression and multimorbidity in older Korean adults.

Furthermore, although gender differences related to depression and multimorbidity have been observed separately, few studies have examined gender differences related to the association between depression and multimorbidity together. In an epidemiological study of gender differences in depression among older adults, 69 (81%) out of 85 reports found that women were more likely to be depressed than men [16]. The proportion of women with multimorbidity is also higher than that of men [17]. Moreover, gender differences have been observed in terms of the types of common comorbid chronic conditions affecting men and women (men: hypertension with cancer and/or arrhythmia; women: hypertension with arthritis and/or hyperlipidemia) [18]. Gender differences related to depression and multimorbidity, therefore, are likely to be reflected in their association; this possibility should not be overlooked.

In this study, we examined the association between depression and multimorbidity with stratification by gender. Furthermore, we examined which combinations of chronic conditions had the most substantial effects on depression in men and women.

## MATERIALS AND METHODS

### Database and study population

The National Survey of Older Koreans (NSOK) is a nationally representative, cross-sectional survey conducted by the Korea Institute for Health and Social Affairs. These data contain information on the living conditions, needs, and characteristics of older Koreans and, per legislative requirements, they have been collected every 3 years since 2008 [19]. We restricted the analysis to the 2011-2017 NSOK dataset since previous data did not include information about exercise habits and insurance type, which were covariates in this study.

Among the 31,744 respondents, we excluded participants diagnosed with depression (n=979) and who answered “preschool children under 10 years of age” to the question about education level (n=1). Additionally, we excluded participants who had missing values for depression score (n = 453), frequency of contact (n=270), exercise time (n=2), and frequency of drinking (n=1). As a result, the total sample included in this study contained 30,138 participants.

### Depression

Depression was measured using the Korean version of the shortform Geriatric Depression Scale (SGDS-K). The SGDS-K comprises 15 items to identify depression symptoms experienced during the previous week. Five of these items are scored from 0 (yes) to 1 (no), and the remaining items are reverse-scored. Total possible scores range from 0 to 15. A higher score indicates more depressive symptoms, and according to the tool’s developer [20], a score of 8 or above indicates depression.

### Multimorbidity

The NSOK surveys 32 chronic diseases, but only 8 were considered in this study: arthritis, diabetes mellitus, heart disease (angina pectoris, myocardial infarction, other heart problems), hypertension, pulmonary disease (chronic bronchitis, emphysema, asthma), cancer, stroke, and osteoporosis. The criteria for choosing diseases were determined based on a previous study [15]. All diseases were self-reported by participants who had been diagnosed by a doctor at least 3 months prior to the survey. Participants were considered to have multimorbidity if they had more than 2 of the 8 chronic diseases simultaneously.

### Covariates

We considered the factors that could potentially confound the association between multimorbidity and depression. The covariates were as follows: gender (men, women), age (65-69, 70-74, 75-79, ≥ 80 years), marital status (married, single/divorced/separated/widowed), living arrangement (living alone, living with someone), education level (≤ primary school, middle school, high school, ≥ college), insurance type (National Health Insurance, Medical Aid), current smoking, lack of exercise, high-risk alcohol drinking, restrictions on activities of daily living (ADL), frequency of contact with people (hardly ever, at least once per year), other chronic conditions, and year.

In the NSOK data, several factors indicated socioeconomic status, including household income and insurance type. When household income was included as a covariate, as in other studies, the 95% confidence interval (CI) corresponding to the frequency of the contact variable was significant but abnormally wide. Based on evidence suggesting that insurance type affects depression among older adults [21], we included insurance type as a covariate instead of household income.

Health behaviors such as smoking, lack of exercise, and high-risk alcohol drinking were assessed based on whether they currently smoked, exercised more than 150 minutes per week, and consumed ≥ 7 (men) or ≥ 5 (women) drinks in a 2-week period [22]. When setting the standard for lack of exercise, since the frequency of exercise in the 2011 NSOK was recorded on a monthly basis, we divided the totals by 4 to measure this parameter in terms of weeks.

A previous study [15] designated the degree of assistance needed as a covariate based on a report [14] that found that older adults living in residential care facilities tended to feel more depressed than community-dwelling older adults. However, we used restrictions on ADL as a covariate instead of the degree of assistance needed since it included those who did not receive formal assistance despite difficulties with ADL. Restrictions on ADL were determined based on whether the respondent answered that he or she needed partial or complete assistance at least once to questions about ADL and instrumental ADL. This study also controlled for social support that negatively affected depression and multimorbidity [23] based on whether the participants had contact with family, friends, or acquaintances at least once in the past year.

In addition to the 8 chronic conditions that were assessed for multimorbidity, any other chronic conditions were assessed by grouping them into a single variable [24]. Since individuals’ living habits changed over time, chronic conditions surveyed on a yearly basis were continually added, and these additional chronic conditions could also affect depression.

### Statistical analysis

In the primary analyses, we investigated the association between depression and multimorbidity. First, we examined the frequency of participants’ characteristics related to depression. The p-values, which were calculated using the chi-square test, were considered to indicate statistical significance when below 0.05. Multiple logistic regression was performed to estimate the odds ratios (ORs) and 95% CIs for the effect of multimorbidity on depression. Moreover, we conducted subgroup analysis by characteristics (gender, age, marital status, living arrangement, education, insurance type, current smoking, lack of exercise, high-risk alcohol drinking, restrictions on ADL, frequency of contact, and presence of other chronic conditions) and tested for interaction effects. In the secondary analyses, we examined the association between depression and combinations of chronic conditions. First, we examined the number of chronic conditions (ranging from 0 to 8) and their associations with depression. Then, we tested various combinations of the 8 chronic conditions (arthritis, diabetes, heart disease, hypertension, pulmonary disease, cancer, stroke, and osteoporosis) to determine which combinations were most closely associated with depression.

### Ethics statement

All participants in this study were protected according to the tenets of the Declaration of Helsinki. The NSOK was approved by the National Statistical Office (approval No. 11771) before each survey was conducted. In 2013, it became mandatory to establish an institutional review board (IRB) under the Bioethics and Safety Act, and the 2017 NSOK was approved by the IRB of the Korean Institute for Health and Social Affairs (IRB No.17-034-00). All participants provided informed consent before enrollment, and all participants’ records were anonymously provided to researchers who agreed to comply with the management rules.

## RESULTS

The participants’ general characteristics are reported in Table 1. The results show that 37.3% of men and 59.2% of women reported multimorbidity. Of them, 31.0% of men and 36.2% of women experienced depression.

Table 2 shows the associations between depression and the participants’ characteristics. In both men and women, those with multimorbidity had a higher risk of depression than those without chronic conditions (men: OR, 2.13; 95% CI, 1.88 to 2.42; women: OR, 1.90; 95% CI, 1.68 to 2.15). The difference in risk among men was greater than the difference in risk among women. Additionally, a greater number of chronic conditions corresponded to a higher risk of depression in men than in women (Supplementary Material 1).

Table 3 presents the stratified analyses according to age and other chronic conditions. Young-old women with multimorbidity had higher levels of depression than old-old women. However, no interaction effect was observed in men. Individuals who reported having any of the 8 chronic conditions had higher levels of depression than their counterparts among both men and women. The results are presented in Supplementary Material 2.

We also examined comorbidities with the 8 chronic conditions and their associations with depression (Table 4). For single chronic conditions, there was a significant association, in descending order, between depression and cancer, stroke, pulmonary disease, arthritis, diabetes, and heart disease. For multimorbidity, combinations that included pulmonary disease, stroke, or cancer were associated with a high level of depression. The results are shown in Supplementary Material 3.

Gender differences related to comorbid chronic conditions were observed (Supplementary Material 4). Among the individual chronic conditions, diabetes (men: OR, 1.16; 95% CI, 0.85 to 1.58; women: OR, 1.67; 95% CI, 1.25 to 2.24) and heart disease (men: OR, 1.14; 95% CI, 0.83 to 1.58; women: OR, 1.74; 95% CI, 1.24 to 2.46) were associated with depression in women only, while pulmonary disease (men: OR, 1.94; 95% CI, 1.41 to 2.68; women: OR, 1.30; 95% CI, 0.78 to 2.18) and osteoporosis (men: OR, 2.53; 95% CI, 1.34 to 4.77; women: OR, 1.02; 95% CI, 0.78 to 1.34) were associated with depression in men only. For multimorbidity, the association between heart disease and depression differed between men and women based on the number of comorbid chronic conditions.

## DISCUSSION

Among the 30,138 older Korean adults, multimorbidity was associated with a high-risk of depression. Moreover, the effects of multimorbidity differed by gender. Compared to older Korean adults with no chronic conditions, men with multimorbidity had a 2.1-fold higher risk, and women with multimorbidity had a 1.9-fold higher risk of depression. Surprisingly, multimorbidity, compared to having no chronic conditions, corresponded to a higher risk of depression in men than in women. This is inconsistent with previous studies that found that women were more likely to have more chronic conditions [17,25] and a higher risk of depression than men [16].

Subgroup analysis revealed an interaction effect related to age. In particular, age moderated the association between multimorbidity and depression in women. In particular, older women with multimorbidity had a higher risk of depression than those without chronic conditions. However, young-old women with multimorbidity had a greater risk of depression than old-old women with multimorbidity. This differed from the results of a previous study that found that older people are at a higher risk of depression after examining only the association between depression and age. The reason for this result may be the effect of age on the association between self-rated health (SRH) and depression. According to a previous study [26], the effect of SRH on depressive symptoms was stronger among those aged 65-79 years compared to those aged 80 years or older. One explanation is that old-old people tend to cognitively adapt to changes in their health. They tend to compare their health to that of others their age, and since most older people experience many chronic conditions as they get older, they can more positively accept changes in health status, such as the number of chronic conditions, compared to young-old people [27,28]. A secondary explanation may be that young-old people consider their health to be their functional status, reflecting their ability to participate in different activities [29]. Therefore, young-old people can react sensitively to changes in health, which can lead to depressive symptoms. For these reasons, mental health care among young-old people with multimorbidity must be monitored closely.

In our secondary analyses, we found a positive association between depression and the number of chronic conditions. The frequency of the same number of chronic conditions was higher among women than among men, and depression compared to frequency was higher for men than for women, indicating the presence of gender differences.

Analyses of specific comorbid chronic conditions associated with depression were conducted both overall and after stratifying by gender. The most important finding in this analysis was the independent effect of pulmonary disease on depression. A previous study [30] in which 5 chronic conditions were combined suggested that people with only pulmonary disease were not at risk for depression compared to those with other chronic conditions. Unexpectedly, in our study, individuals diagnosed with only pulmonary disease reported the third-highest depression level (OR, 1.76) among the 8 chronic conditions. Consistent with these findings, combinations that included pulmonary disease (e.g., pulmonary disease-stroke, diabetes-pulmonary disease, hypertension-pulmonary disease-cancer, diabetes-hypertension-pulmonary disease-cancer) were associated with a high risk of depression. Our study showed that people with ADL restrictions had a greater risk of depression. In previous studies, pulmonary diseases such as chronic obstructive pulmonary disease and chronic bronchitis were found to restrict ADL [31]. In addition, asthma is highly associated with depression in individuals [32]. Thus, improving ADL was a mediator for the association between asthma and depression [32].

Our findings on comorbidities involving stroke were also significant. Individuals with a history of stroke had a 2.81 times higher likelihood of depression than those without chronic conditions, which was the second-highest level of depression among the 8 chronic conditions. In addition, combinations of pulmonary disease with stroke, diabetes with stroke, and arthritis with stroke had the highest risks of depression among the other 2-disease combinations. Depression is more common in stroke patients than in people with similar physical disabilities caused by orthopedic diseases such as severe arthritis [33]. A previous study found that people with stroke had to improve their ADL for treatment through initiatives such as “plan, do, check, and action” nursing [34]. According to this study, stroke affected the limits of an individual’s ADL [34]. In addition, many studies have shown that improved ADL has a preventive effect on depression [32,35].

The strong effect of cancer on depression must also be emphasized. The independent effect of cancer on depression showed the highest risk (OR, 3.04) among the 8 chronic conditions. Cancer is a life-threatening disease [36]; thus, a cancer diagnosis can cause a greater sense of distress than non-neoplastic diseases with poorer prognoses in patients [37], and mental distress in cancer patients experienced for sustained periods of time may lead to depression [38]. One study found an increased number of comorbidities to be associated with a higher level of depression among older adults with cancer [39]. However, in this study, some combinations of conditions (such as arthritis and cancer; heart disease and cancer; hypertension and cancer; and arthritis, hypertension, and cancer) had weaker associations with depression than in patients with cancer only. Therefore, cancer patients should be treated based on the expected risk of depression associated with their chronic conditions in addition to cancer rather than simply based on a patient’s total number of comorbidities.

The analysis of gender differences yielded results that contradicted the widespread assumption in the medical field that morbidity related to heart disease is more common among men and morbidity related to osteoporosis is more common among women. Based on this set of assumptions, most past studies of osteoporosis have focused on postmenopausal women. However, according to an innovative study that explored differences according to gender, morbidity and mortality related to major osteoporosis fractures were significantly higher among men than among women [40]. Concerning heart disease, women were found to have a worse prognosis than men [41]. These results could also be associated with mental health.

Another gender difference was observed in the effect of heart disease on depression. Gender differences were observed related to the number of chronic conditions. In women, heart disease was found to have an effect on depression when accompanied by 1 or 2 chronic conditions. Heart disease also had a significant independent effect on depression in women. In addition, the combination of heart disease and pulmonary disease had the strongest association with depression among all other possible comorbidities related to either condition. However, when women had 3 or more chronic conditions, combinations that included cancer, stroke, and osteoporosis (e.g., arthritis, cancer, and osteoporosis, or arthritis, diabetes, stroke, and osteoporosis) were associated with higher levels of depression. In contrast, in men, the effects of heart disease on depression were observed when they had 3 or more chronic conditions. Heart disease did not independently affect depression in men like it did with diabetes and hypertension. However, when men had 3 or more chronic conditions, combinations that included heart disease (e.g., diabetes, heart disease, and pulmonary disease; or heart disease, hypertension, pulmonary disease, and stroke) significantly increased the risk of depression. Older adults with heart disease may become depressed due to a fear of death [42], and women with heart disease actually have a higher mortality rate than men with heart disease [43]. Therefore, unlike in men, depression can be assumed to have a stronger association with heart disease in women even without additional comorbidities.

This study had several limitations. The sample was too small to yield significant results on the association between depression and different comorbid chronic conditions. Although all individuals with particular combinations of conditions (such as arthritis–heart disease–cancer–osteoporosis, diabetes–stroke–osteoporosis) had symptoms of depression, the results were insignificant due to the small sample size. Thus, other comorbid chronic conditions that might have a great impact on depression could not be considered. Second, depression may have been underestimated since people who had been diagnosed with depression were excluded from the study. Third, we determined the presence of chronic conditions using self-reports. This method can underrepresent the number of people affected by chronic conditions that they inadvertently overlooked or had not been diagnosed with by a doctor, such as hypertension and diabetes [44]. Fourth, we could not evaluate the effect of other chronic conditions (such as chronic obstructive pulmonary disease) known to be associated with depression [45] since the types of chronic conditions investigated were restricted. Fifth, in previous studies, in addition to the number of chronic conditions, the severity of chronic conditions also affected depression [46-48]. The severity of chronic conditions such as asthma, stroke, and diabetes increases the risk of depression [46-48]. However, the severity of chronic conditions was not analyzed in this study. Thus, this should be included in future studies for accurate analyses. Finally, the cross-sectional data only measured the prevalence of depression and did not determine the onset of depression. Therefore, a causal association cannot be determined. However, we excluded those who had previously been diagnosed with depression. Therefore, the results indicated that multimorbidity might increase depression. In other words, although we found that chronic disease diagnoses were associated with elevated levels of depression, the diagnoses could not be determined to have caused elevated depression [24].

Despite the aforementioned limitations, this study has several strengths. First, to the best of our knowledge, this is the first study to analyze comorbid chronic conditions using the multimorbidity index from previous studies [30,49] and complements the extant body of literature that included only a small number of chronic conditions. Although a previous study [15] examined the association between multimorbidity and depression in terms of the same 8 chronic conditions included in this study, it did not evaluate different combinations of these conditions. Second, the effects of gender were not overlooked since men and women were classified and analyzed separately. Numerous studies have examined the impact of gender on multimorbidity and depression, but few have investigated how the association between multimorbidity and depression differs by gender.

In conclusion, we found that multimorbidity, and specifically the number of chronic conditions, were strongly associated with depression in older Korean adults. In addition, men with multimorbidity reported higher levels of depression than women with multimorbidity. Gender differences were also observed in terms of comorbid chronic conditions. Using the present study as a stepping stone, future studies can accurately interpret patients’ depression levels based on combinations of chronic conditions. Based on our findings, gender-specific prevention methods should be used to provide treatment to individuals with multimorbidity who experience depressive symptoms.

## Figures and Tables

**Table 1.** General characteristics of study participants according to depression by gender

Characteristics		Total (n=30,138)			p-value	Men (n=12,298)			p-value	Women (n=17,840)			p-value
		No	Yes	Total, %		No	Yes	Total, %		No	Yes	Total, %	
No. of chronic conditions													
	0	4,663 (84.4)	860 (15.6)	18.3	<0.001	2,805 (86.1)	454 (13.9)	26.5	<0.001	1,858 (82.1)	406 (17.9)	12.7	<0.001
	1	7,375 (77.9)	2,089 (22.1)	31.4		3,600 (81.0)	847 (19.1)	36.2		3,775 (75.2)	1,242 (24.8)	28.1	
	≥2	9,902 (65.4)	5,249 (34.6)	50.3		3,167 (69.0)	1,425 (31.0)	37.3		6,735 (63.8)	3,824 (36.2)	59.2	
Gender													
	Men	9,572 (77.8)	2,726 (22.2)	40.8	<0.001	-	-	-	-	-	-	-	-
	Women	12,368 (69.3)	5,472 (30.7)	59.2		-	-	-		-	-	-	
Age (yr)													
	65-69	6,298 (81.4)	1,443 (18.6)	25.7	<0.001	2,729 (84.0)	521 (16.0)	26.4	<0.001	3,569 (79.5)	922 (20.5)	25.2	<0.001
	70-74	6,637 (75.3)	2,173 (24.7)	29.2		2,991 (80.0)	748 (20.0)	30.4		3,646 (71.9)	1,425 (28.1)	28.4	
	75-79	5,270 (69.0)	2,367 (31.0)	25.3		2,342 (75.1)	777 (24.9)	28.4		2,928 (64.8)	1,590 (35.2)	25.3	
	≥80	3,735 (62.8)	2,215 (37.2)	19.7		1,510 (69.0)	680 (31.1)	17.8		2,225 (59.2)	1,535 (40.8)	21.1	
Marital status													
	Single, divorced, separated, or widowed	7,340 (64.6)	4,028 (35.4)	37.7	<0.001	1,053 (67.4)	509 (32.6)	12.7	<0.001	6,287 (64.1)	3,519 (35.9)	55.0	<0.001
	Married	14,600 (77.8)	4,170 (22.2)	62.3		8,519 (79.4)	2,217 (20.7)	87.3		6,081 (75.7)	1,953 (24.3)	45.0	
Living arrangement													
	Living alone	4,745 (64.2)	2,648 (35.8)	24.5	<0.001	778 (66.4)	393 (33.6)	9.5	<0.001	3,967 (63.8)	2,255 (36.2)	34.9	<0.001
	Living with someone	17,195 (75.6)	5,550 (24.4)	75.5		8,794 (79.0)	2,333 (21.0)	90.5		8,401 (72.3)	3,217 (27.7)	65.1	
Education level													
	≤Primary school	13,600 (67.5)	6,535 (32.5)	66.8	<0.001	4,143 (71.6)	1,643 (28.4)	47.1	<0.001	9,457 (65.9)	4,892 (34.1)	80.4	<0.001
	Middle school	3,285 (80.9)	778 (19.2)	13.5		1,856 (80.4)	454 (19.7)	18.8		1,429 (81.5)	324 (18.5)	9.8	
	High school	3,435 (83.3)	688 (16.7)	13.7		2,300 (83.1)	468 (16.9)	22.5		1,135 (83.8)	220 (16.2)	7.6	
	≥College	1,620 (89.2)	197 (10.8)	6.0		1,273 (88.8)	161 (11.2)	11.7		347 (90.6)	36 (9.4)	2.2	
Insurance type													
	National Health Insurance	20,872 (74.6)	7,091 (25.4)	92.8	<0.001	9,226 (79.4)	2,396 (20.6)	94.5	<0.001	11,646 (71.3)	4,695 (28.7)	91.6	<0.001
	Medical Aid	1,068 (49.1)	1,107 (50.9)	7.2		346 (51.2)	330 (48.8)	5.5		722 (48.2)	777 (51.8)	8.4	
Current smoking													
	No	19,539 (73.0)	7,221 (27.0)	88.8	0.017	7,501 (78.8)	2,018 (21.2)	77.4	<0.001	12,038 (69.8)	5,203 (30.2)	96.6	<0.001
	Yes	2,401 (71.1)	977 (28.9)	11.2		2,071 (74.5)	708 (25.5)	22.6		330 (55.1)	269 (44.9)	3.4	
Lack of exercise													
	No	10,405 (82.1)	2,276 (18.0)	42.1	<0.001	5,281 (85.0)	932 (15.0)	50.5	<0.001	5,124 (79.2)	1,344 (20.8)	36.3	<0.001
	Yes	11,535 (66.1)	5,922 (33.9)	57.9		4,291 (70.5)	1,794 (29.5)	49.5		7,244 (63.7)	4,128 (36.3)	63.7	
High-risk alcohol drinking													
	No	20,635 (72.3)	7,899 (27.7)	94.7	<0.001	8,314 (77.2)	2,450 (22.8)	87.5	<0.001	12,321 (69.3)	5,449 (30.7)	99.6	0.691
	Yes	1,305 (81.4)	299 (18.6)	5.3		1,258 (82.0)	276 (18.0)	12.5		47 (67.1)	23 (32.9)	0.4	
Restriction on activities of daily living													
	No	19,032 (77.9)	5,395 (22.1)	81.1	<0.001	8,868 (81.9)	1,962 (18.1)	88.1	<0.001	10,164 (74.8)	3,433 (25.3)	76.2	<0.001
	Yes	2,908 (50.9)	2,803 (49.1)	19.0		704 (48.0)	764 (52.0)	11.9		2,204 (51.9)	2,039 (48.1)	23.8	
Frequency of contact with people													
	Hardly ever	13 (33.3)	26 (66.7)	0.1	<0.001	8 (40.0)	12 (60.0)	0.2	<0.001	5 (26.3)	14 (73.7)	0.1	<0.001
	At least once	21,927 (72.9)	8,172 (27.2)	99.9		9,564 (77.9)	2,714 (22.1)	99.8		12,363 (69.4)	5,458 (30.6)	99.9	
Other chronic conditions^1^													
	No	9,100 (79.7)	2,317 (20.3)	37.9	<0.001	4,441 (83.7)	865 (16.3)	43.2	<0.001	4,659 (76.2)	1,452 (23.8)	34.3	<0.001
	Yes	12,840 (68.6)	5,881 (31.4)	62.1		5,131 (73.4)	1,861 (26.6)	56.9		7,709 (65.7)	4,020 (34.3)	65.8	
Year													
	2011	7,331 (70.1)	3,127 (29.9)	34.7	<0.001	3,186 (75.6)	1,030 (24.4)	34.3	<0.001	4,145 (66.4)	2,097 (33.6)	35.0	<0.001
	2014	6,826 (68.9)	3,089 (31.2)	32.9		3,089 (75.0)	1,029 (25.0)	33.5		3,737 (64.5)	2,060 (35.5)	32.5	
	2017	7,783 (79.7)	1,982 (20.3)	32.4		3,297 (83.2)	667 (16.8)	32.2		4,486 (77.3)	1,315 (22.7)	32.5	

Values are presented as number (%).

1 Other chronic conditions: hyperlipidemia, thyroid disease, low back pain, sciatica, pulmonary tuberculosis, tuberculosis, cataract, glaucoma, chronic otitis media, stomach ulcer, duodenal ulcer, hepatitis, liver cirrhosis, chronic kidney disease, prostatic hypertrophy, urinary incontinence, venereal disease, anemia, skin disease, dementia, fracture, dislocation, sequela, insomnia, Parkinson’s disease, and others.

**Table 2.** The associations between depression and the characteristics of study participants by gender^1^

Variable		Men	Women
No. of chronic conditions			
	0	1.00 (reference)	1.00 (reference)
	1	1.32 (1.15, 1.50)	1.30 (1.14, 1.48)
	≥2	2.13 (1.88, 2.42)	1.90 (1.68, 2.15)
	p for trend	<0.001	<0.001
Age (yr)			
	65-69	1.00 (reference)	1.00 (reference)
	70-74	1.13 (0.99, 1.29)	1.18 (1.07, 1.31)
	75-79	1.39 (1.22, 1.59)	1.37 (1.23, 1.52)
	≥80	1.42 (1.23, 1.65)	1.39 (1.24, 1.55)
	p for trend	<0.001	<0.001
Marital status			
	Single, divorced, separated, or widowed	1.27 (1.01, 1.60)	1.17 (1.07, 1.29)
	Married	1.00 (reference)	1.00 (reference)
Living arrangement			
	Living alone	1.36 (1.05, 1.76)	1.09 (0.99, 1.19)
	Living with someone	1.00 (reference)	1.00 (reference)
Education level			
	≤Primary school	2.30 (1.91, 2.77)	3.20 (2.23, 4.60)
	Middle school	1.77 (1.44, 2.18)	2.16 (1.48, 3.17)
	High school	1.56 (1.28, 1.92)	1.92 (1.30, 2.84)
	≥College	1.00 (reference)	1.00 (reference)
Insurance type			
	National Health Insurance	1.00 (reference)	1.00 (reference)
	Medical Aid	2.50 (2.10, 2.99)	2.11 (1.88, 2.37)
Current smoking			
	No	1.00 (reference)	1.00 (reference)
	Yes	1.26 (1.13, 1.41)	1.59 (1.33, 1.90)
Lack of exercise			
	No	1.00 (reference)	1.00 (reference)
	Yes	1.83 (1.66, 2.02)	1.70 (1.57, 1.83)
High-risk alcohol drinking			
	No	1.00 (reference)	1.00 (reference)
	Yes	0.84 (0.72, 0.97)	1.14 (0.67, 1.93)
Restriction on activities of daily living			
	No	1.00 (reference)	1.00 (reference)
	Yes	3.54 (3.12, 4.02)	2.30 (2.12, 2.50)
Frequency of contact with people			
	Hardly ever	2.99 (1.11, 8.06)	2.79 (0.94, 8.26)
	At least once	1.00 (reference)	1.00 (reference)
Other chronic conditions			
	No	1.00 (reference)	1.00 (reference)
	Yes	1.53 (1.39, 1.69)	1.48 (1.37, 1.60)
Year			
	2011	1.90 (1.69, 2.15)	2.02 (1.85, 2.21)
	2014	2.00 (1.77, 2.26)	2.28 (2.08, 2.49)
	2017	1.00 (reference)	1.00 (reference)

Values are presented as odds ratio (95% confidence interval).

1 Adjusted for age, marital status, living arrangement, education level, insurance type, current smoking, lack of exercise, high-risk alcohol drinking, restriction on activities of daily living, frequency of contact with people, other chronic conditions, and year.

**Table 3.** Subgroup analysis of the association between depression and multimorbidity^1^

Variables	Men			p-value^2^	Women			p-value^2^
	No. of chronic conditions				No. of chronic conditions			
	0	1	≥2		0	1	≥2	
Age (yr)				0.098				0.009
65-69	1.00 (reference)	1.58 (1.20, 2.10)	2.56 (1.95, 3.36)		1.00 (reference)	1.43 (1.09, 1.87)	2.31 (1.79, 2.98)	
70-74	1.00 (reference)	1.34 (1.05, 1.72)	2.14 (1.69, 2.71)		1.00 (reference)	1.54 (1.19, 2.01)	2.13 (1.67, 2.71)	
75-79	1.00 (reference)	1.18 (0.91, 1.53)	2.20 (1.72, 2.82)		1.00 (reference)	0.94 (0.71, 1.24)	1.56 (1.22, 2.01)	
≥80	1.00 (reference)	1.16 (0.88, 1.53)	1.61 (1.22, 2.11)		1.00 (reference)	1.27 (0.97, 1.65)	1.58 (1.24, 2.02)	
Other chronic conditions				0.006				0.001
No	1.00 (reference)	1.63 (1.32, 2.02)	2.66 (2.16, 3.28)		1.00 (reference)	1.44 (1.16, 1.79)	2.41 (1.97, 2.94)	
Yes	1.00 (reference)	1.12 (0.94, 1.33)	1.82 (1.55, 2.14)		1.00 (reference)	1.17 (0.99, 1.39)	1.61 (1.38, 1.89)	

Values are presented as odds ratio (95% confidence interval).

1 Adjusted for age, marital status, living arrangement, education level, insurance type, current smoking, lack of exercise, high-risk alcohol drinking, restriction on activities of daily living, frequency of contact with people, other chronic conditions, and year.

2 Defined as the p-value of interactions.

**Table 4.** Associations between various combinations of chronic conditions and depression (0-2)^1^

Combinations of chronic conditions		Depression		
		Yes, n (%)	Total, n	OR (95% CI)
Zero		860 (15.6)	5,523	1.00 (reference)
One				
	Arthritis	650 (29.1)	2,236	1.55 (1.37, 1.76)
	Diabetes	143 (20.0)	716	1.41 (1.14, 1.73)
	Heart disease	116 (22.7)	511	1.39 (1.10, 1.76)
	Hypertension	793 (17.0)	4,678	1.04 (0.93, 1.17)
	Pulmonary disease	92 (29.9)	308	1.76 (1.35, 2.31)
	Cancer	105 (34.9)	301	3.04 (2.33, 3.96)
	Stroke	79 (38.4)	206	2.81 (2.07, 3.83)
	Osteoporosis	111 (21.9)	508	1.18 (0.93, 1.50)
Two				
	Arthritis+diabetes	103 (31.7)	325	2.01 (1.55, 2.60)
	Arthritis+heart disease	97 (37.2)	261	2.14 (1.62, 2.82)
	Arthritis+hypertension	799 (30.4)	2,628	1.61 (1.42, 1.81)
	Arthritis+pulmonary disease	49 (38.6)	127	2.26 (1.54, 3.31)
	Arthritis+cancer	24 (30.8)	78	1.61 (0.95, 2.70)
	Arthritis+stroke	46 (54.8)	84	4.15 (2.59, 6.63)
	Arthritis+osteoporosis	252 (33.3)	757	1.66 (1.38, 1.99)
	Diabetes+heart disease	35 (27.1)	129	1.89 (1.24, 2.88)
	Diabetes+hypertension	437 (23.9)	1,828	1.57 (1.37, 1.81)
	Diabetes+pulmonary disease	12 (50.0)	24	4.29 (1.80, 10.21)
	Diabetes+cancer	24 (40.7)	59	4.11 (2.33, 7.16)
	Diabetes+stroke	30 (52.6)	57	4.92 (2.78, 8.81)
	Diabetes+osteoporosis	17 (25.8)	66	1.46 (0.81, 2.65)
	Heart disease+hypertension	160 (22.4)	713	1.43 (1.17, 1.75)
	Heart disease+pulmonary disease	20 (47.6)	42	3.38 (1.78, 6.50)
	Heart disease+cancer	7 (28.0)	25	2.15 (0.83, 5.60)
	Heart disease+stroke	11 (36.7)	30	1.81 (0.82, 4.03)
	Heart disease+osteoporosis	19 (34.6)	55	1.83 (1.02, 3.30)
	Hypertension+pulmonary disease	62 (32.3)	192	2.00 (1.43, 2.78)
	Hypertension+cancer	48 (29.5)	163	2.25 (1.57, 3.25)
	Hypertension+stroke	195 (38.0)	513	2.53 (2.06, 3.12)
	Hypertension+osteoporosis	171 (29.3)	583	1.50 (1.22, 1.85)
	Pulmonary disease+cancer	6 (42.9)	14	3.90 (1.28, 11.98)
	Pulmonary disease+stroke	10 (66.7)	15	6.88 (2.19, 20.99)
	Pulmonary disease+ osteoporosis	10 (34.5)	29	2.26 (1.01, 5.12)
	Cancer+stroke	5 (55.6)	9	4.19 (0.97, 18.17)
	Cancer+osteoporosis	8 (36.4)	22	2.84 (1.10, 7.35)
	Stroke+osteoporosis	4 (26.7)	15	1.13 (0.33, 3.90)

OR, odds ratio; CI, confidence interval.

1 Adjusted for gender, age, marital status, living arrangement, education, type of insurance, current smoking, lack of exercise, high-risk alcohol drinking, restriction on activities of daily living, frequency of contact with people, other chronic conditions, and year.

## SUPPLEMENTARY MATERIALS

Supplementary Material 1. Depression based on the number of chronic conditions by gender

Supplementary Material 2. A subgroup analysis of the association between depression and multimorbidity

Supplementary Material 3. The association between chronic conditions combinations and depression

Supplementary Material 4. The association between chronic conditions combinations and depression by gender
