# Supplementary material for: Gender differences in the association between multimorbidity and depression in older Korean adults: an analysis of data from the National Survey of Older Koreans (2011-2017)
Source: Epidemiol Health. 2022 May 24;44:e2022049. doi: 10.4178/epih.e2022049 (PMC9714839; doi:10.4178/epih.e2022049)
Supplement: Supplementary Material 1. — Depression based on the number of chronic conditions by gender [file epih-44-e2022049-suppl1.docx]

| Supplementary Material 1. Depression based on the number of chronic conditions by gender | | | | | | | | | | | | | | | | |
| --- | --- | --- | --- | --- | --- | --- | --- | --- | --- | --- | --- | --- | --- | --- | --- | --- |
|  |  |  |  |  |  |  |  |  |  |  |  |  |  |  |  |  |
| Number of  chronic conditions | | Men | | | | | | |  | Women | | | | | | |
|  |  | Depression | | | | | | |  | Depression | | | | | | |
|  |  | Yes | | Total | | OR | 95% CI | |  | Yes | | Total | | OR | 95% CI | |
|  |  | N (%) | | N | |  |  |  |  | N (%) | | N | |  |  |  |
| 0 | | 454 (13.9) | | 3259 | | 1.00 |  | |  | 406 (17.9) | | 2264 | | 1.00 |  | |
| 1 | | 847 (19.1) | | 4447 | | 1.32 | 1.16 | 1.51 |  | 1242 (24.8) | | 5017 | | 1.31 | 1.14 | 1.49 |
| 2 | | 854 (27.8) | | 3075 | | 1.92 | 1.68 | 2.20 |  | 1807 (31.3) | | 5768 | | 1.64 | 1.44 | 1.87 |
| 3 | | 404 (34.1) | | 1184 | | 2.26 | 1.91 | 2.69 |  | 1294 (39.0) | | 3319 | | 2.07 | 1.80 | 2.37 |
| 4 | | 135 (49.3) | | 274 | | 4.18 | 3.15 | 5.53 |  | 559 (47.0) | | 1189 | | 2.67 | 2.25 | 3.15 |
| 5 | | 32 (59.3) | | 54 | | 6.18 | 3.40 | 11.25 |  | 141 (57.3) | | 246 | | 3.45 | 2.57 | 4.62 |
| 6 | | 0 (0.0) | | 5 | | - | - | - |  | 21 (63.6) | | 33 | | 3.71 | 1.73 | 7.94 |
| 7 | | - | | - | | - | - | - |  | 2 (50.0) | | 4 | | 1.79 | 0.15 | 20.96 |
| 8 | | - | | - | | - | - | - |  | - | | - | | - | - | - |
| Adjusted for age, marital status, living arrangement, education, type of insurance, current smoking, lack of exercise, high-risk alcohol drinking, restriction on activities of daily living, frequency of contact with people, other chronic conditions, and year. | | | | | | | | | | | | | | | | |
