# Supplementary material for: Gender differences in the association between multimorbidity and depression in older Korean adults: an analysis of data from the National Survey of Older Koreans (2011-2017)
Source: Epidemiol Health. 2022 May 24;44:e2022049. doi: 10.4178/epih.e2022049 (PMC9714839; doi:10.4178/epih.e2022049)
Supplement: Supplementary Material 2. — A subgroup analysis of the association between depression and multimorbidity [file epih-44-e2022049-suppl2.docx]

| Supplementary Material 2. A subgroup analysis of the association between depression and multimorbidity | | | | | | | | | | | | | | | | | |
| --- | --- | --- | --- | --- | --- | --- | --- | --- | --- | --- | --- | --- | --- | --- | --- | --- | --- |
|  | | | | | | | | | | | | | | | | | |
| Variable | | Men | | | | | | | p* | Women | | | | | | | p* |
|  |  | Number of chronic conditions | | | | | | |  | Number of chronic conditions | | | | | | |  |
|  |  | 0 | 1 | | | 2+ | | |  | 0 | 1 | | | 2+ | | |  |
|  |  | OR | OR | 95% CI | | OR | 95% CI | |  | OR | OR | 95% CI | | OR | 95% CI | |  |
| Age | |  |  |  |  |  |  |  |  |  |  |  |  |  |  |  |  |
|  | 65-69 | 1.00 | 1.58 | 1.20 | 2.10 | 2.56 | 1.95 | 3.36 | 0.0978 | 1.00 | 1.43 | 1.09 | 1.87 | 2.31 | 1.79 | 2.98 | 0.0088 |
|  | 70-74 | 1.00 | 1.34 | 1.05 | 1.72 | 2.14 | 1.69 | 2.71 |  | 1.00 | 1.54 | 1.19 | 2.01 | 2.13 | 1.67 | 2.71 |  |
|  | 75-79 | 1.00 | 1.18 | 0.91 | 1.53 | 2.20 | 1.72 | 2.82 |  | 1.00 | 0.94 | 0.71 | 1.24 | 1.56 | 1.22 | 2.01 |  |
|  | 80+ | 1.00 | 1.16 | 0.88 | 1.53 | 1.61 | 1.22 | 2.11 |  | 1.00 | 1.27 | 0.97 | 1.65 | 1.58 | 1.24 | 2.02 |  |
| Marital status | | |  |  |  |  |  |  |  |  |  |  |  |  |  |  |  |
|  | Single, divorced, separated, widowed | 1.00 | 1.10 | 0.79 | 1.53 | 2.02 | 1.48 | 2.76 | 0.4286 | 1.00 | 1.20 | 1.01 | 1.43 | 1.75 | 1.49 | 2.05 | 0.1381 |
|  | Married | 1.00 | 1.35 | 1.17 | 1.56 | 2.13 | 1.86 | 2.45 |  | 1.00 | 1.45 | 1.18 | 1.79 | 2.12 | 1.75 | 2.58 |  |
| Living arrangment | | | |  |  |  |  |  |  |  |  |  |  |  |  |  |  |
|  | Living alone | 1.00 | 1.24 | 0.85 | 1.81 | 2.17 | 1.51 | 3.11 | 0.9572 | 1.00 | 1.23 | 0.98 | 1.53 | 1.70 | 1.39 | 2.08 | 0.1230 |
|  | Living with someone | 1.00 | 1.32 | 1.14 | 1.52 | 2.12 | 1.85 | 2.42 |  | 1.00 | 1.35 | 1.14 | 1.59 | 2.04 | 1.74 | 2.37 |  |
| Education | |  |  |  |  |  |  |  |  |  |  |  |  |  |  |  |  |
|  | ≤ Primary school | 1.00 | 1.43 | 1.20 | 1.70 | 2.13 | 1.80 | 2.52 | 0.3627 | 1.00 | 1.28 | 1.11 | 1.48 | 1.85 | 1.62 | 2.12 | 0.7784 |
|  | Middle school | 1.00 | 1.14 | 0.83 | 1.57 | 2.03 | 1.50 | 2.75 |  | 1.00 | 1.53 | 0.96 | 2.45 | 2.12 | 1.37 | 3.27 |  |
|  | High school | 1.00 | 1.05 | 0.77 | 1.43 | 1.99 | 1.48 | 2.67 |  | 1.00 | 1.07 | 0.63 | 1.82 | 2.02 | 1.23 | 3.29 |  |
|  | ≥ College | 1.00 | 1.70 | 1.01 | 2.88 | 2.62 | 1.57 | 4.38 |  | 1.00 | 1.72 | 0.49 | 6.01 | 2.27 | 0.67 | 7.61 |  |
| (Continued to next page) | | | | | | | | | | | | | | | | | |

| Supplementary Material 2. Continued | | | | | | | | | | | | | | | | | |
| --- | --- | --- | --- | --- | --- | --- | --- | --- | --- | --- | --- | --- | --- | --- | --- | --- | --- |
|  | | | | | | | | | | | | | | | | | |
| Variable | | Men | | | | | | | p* | Women | | | | | | | p* |
|  |  | Number of chronic conditions | | | | | | |  | Number of chronic conditions | | | | | | |  |
|  |  | 0 | 1 | | | 2+ | | |  | 0 | 1 | | | 2+ | | |  |
|  |  | OR | OR | 95% CI | | OR | 95% CI | |  | OR | OR | 95% CI | | OR | 95% CI | |  |
| Type of insurance | | | |  |  |  |  |  |  |  |  |  |  |  |  |  |  |
|  | National Health Insurance | 1.00 | 1.36 | 1.19 | 1.56 | 2.16 | 1.89 | 2.46 | 0.2310 | 1.00 | 1.35 | 1.18 | 1.56 | 1.97 | 1.73 | 2.24 | 0.0779 |
|  | Medical aid | 1.00 | 0.88 | 0.52 | 1.47 | 1.77 | 1.09 | 2.87 |  | 1.00 | 0.83 | 0.53 | 1.30 | 1.23 | 0.82 | 1.85 |  |
| Current smoking | | |  |  |  |  |  |  |  |  |  |  |  |  |  |  |  |
|  | No | 1.00 | 1.44 | 1.23 | 1.69 | 2.23 | 1.92 | 2.60 | 0.1271 | 1.00 | 1.32 | 1.15 | 1.51 | 1.90 | 1.68 | 2.16 | 0.4383 |
|  | Yes | 1.00 | 1.10 | 0.87 | 1.40 | 1.99 | 1.58 | 2.52 |  | 1.00 | 1.02 | 0.56 | 1.85 | 1.94 | 1.11 | 3.40 |  |
| Lack of exercise | | |  |  |  |  |  |  |  |  |  |  |  |  |  |  |  |
|  | No | 1.00 | 1.32 | 1.06 | 1.63 | 2.37 | 1.93 | 2.90 | 0.3022 | 1.00 | 1.34 | 1.05 | 1.70 | 2.05 | 1.63 | 2.57 | 0.8371 |
|  | Yes | 1.00 | 1.32 | 1.12 | 1.56 | 2.00 | 1.69 | 2.35 |  | 1.00 | 1.28 | 1.09 | 1.50 | 1.84 | 1.59 | 2.13 |  |
| High-risk alcohol drinking | | | | |  |  |  |  |  |  |  |  |  |  |  |  |  |
|  | No | 1.00 | 1.37 | 1.19 | 1.58 | 2.19 | 1.91 | 2.52 | 0.3567 | 1.00 | 1.30 | 1.14 | 1.48 | 1.90 | 1.68 | 2.15 | 0.8610 |
|  | Yes | 1.00 | 1.06 | 0.75 | 1.51 | 1.78 | 1.24 | 2.54 |  | 1.00 | 2.43 | 0.13 | 44.46 | 1.89 | 0.11 | 31.32 |  |
| Restriction on activities of daily living | | | | | |  |  |  |  |  |  |  |  |  |  |  |  |
|  | No | 1.00 | 1.32 | 1.14 | 1.53 | 2.19 | 1.91 | 2.52 | 0.3559 | 1.00 | 1.33 | 1.14 | 1.54 | 1.93 | 1.68 | 2.23 | 0.6328 |
|  | Yes | 1.00 | 1.23 | 0.87 | 1.75 | 1.69 | 1.22 | 2.32 |  | 1.00 | 1.19 | 0.91 | 1.56 | 1.72 | 1.35 | 2.21 |  |
| (Continued to next page) | | | | | | | | | | | | | | | | | |

| Supplementary Material 2. Continued | | | | | | | | | | | | | | | | | |
| --- | --- | --- | --- | --- | --- | --- | --- | --- | --- | --- | --- | --- | --- | --- | --- | --- | --- |
|  | | | | | | | | | | | | | | | | | |
| Variable | | Men | | | | | | | p* | Women | | | | | | | p* |
|  |  | Number of chronic conditions | | | | | | |  | Number of chronic conditions | | | | | | |  |
|  |  | 0 | 1 | | | 2+ | | |  | 0 | 1 | | | 2+ | | |  |
|  |  | OR | OR | 95% CI | | OR | 95% CI | |  | OR | OR | 95% CI | | OR | 95% CI | |  |
| Frequency of contact with people | | | | | |  |  |  |  |  |  |  |  |  |  |  |  |
|  | hardly ever | 1.00 | 16.56 | - | - | - | - | - | 0.7591 | 1.00 | - | - | - | 0.34 | - | - | 0.5222 |
|  | at least once | 1.00 | 1.32 | 1.15 | 1.50 | 2.13 | 1.87 | 2.42 |  | 1.00 | 1.30 | 1.14 | 1.49 | 1.90 | 1.68 | 2.15 |  |
| Other chronic conditions | |  |  |  |  |  |  |  |  |  |  |  |  |  |  |  |  |
|  | No | 1.00 | 1.63 | 1.32 | 2.02 | 2.66 | 2.16 | 3.28 | 0.0063 | 1.00 | 1.44 | 1.16 | 1.79 | 2.41 | 1.97 | 2.94 | 0.0007 |
|  | Yes | 1.00 | 1.12 | 0.94 | 1.33 | 1.82 | 1.55 | 2.14 |  | 1.00 | 1.17 | 0.99 | 1.39 | 1.61 | 1.38 | 1.89 |  |
| *P was defined as interaction p-value. When frequency of contact was 'hardly ever', it was not estimated due to lack of frequency. Adjusted for age, marital status, living arrangement, education, type of insurance, current smoking, lack of exercise, high-risk alcohol drinking, restriction on activities of daily living, frequency of contact with people, other chronic conditions, and year. | | | | | | | | | | | | | | | | | |
