# Supplementary material for: Gender differences in the association between multimorbidity and depression in older Korean adults: an analysis of data from the National Survey of Older Koreans (2011-2017)
Source: Epidemiol Health. 2022 May 24;44:e2022049. doi: 10.4178/epih.e2022049 (PMC9714839; doi:10.4178/epih.e2022049)
Supplement: Supplementary Material 3. — The association between chronic conditions combinations and depression [file epih-44-e2022049-suppl3.docx]

| Supplementary Material 3. The association between chronic conditions combinations and depression | | | | |  |  |  |  |  |
| --- | --- | --- | --- | --- | --- | --- | --- | --- | --- |
|  |  |  |  |  |  |  |  |  |  |
| Chronic condition combinations | | | Depression | | | | | | |
|  |  |  | Yes | | Total | | OR | 95% CI | |
|  |  |  | N (%) | | N | |  |  |  |
| Zero | | | 860 (15.6) | | 5523 | | 1.00 |  | |
| One |  |  |  |  |  |  |  |  |  |
| 1. Arthritis | | | 650 (29.1) | | 2236 | | 1.55 | 1.37 | 1.76 |
| 2. Diabetes | | | 143 (20.0) | | 716 | | 1.41 | 1.14 | 1.73 |
| 3. Heart disease | | | 116 (22.7) | | 511 | | 1.39 | 1.10 | 1.76 |
| 4. Hypertension | | | 793 (17.0) | | 4678 | | 1.04 | 0.93 | 1.17 |
| 5. Pulmonary disease | | | 92 (29.9) | | 308 | | 1.76 | 1.35 | 2.31 |
| 6. Cancer | | | 105 (34.9) | | 301 | | 3.04 | 2.33 | 3.96 |
| 7. Stroke | | | 79 (38.4) | | 206 | | 2.81 | 2.07 | 3.83 |
| 8. Osteoporosis | | | 111 (21.9) | | 508 | | 1.18 | 0.93 | 1.50 |
| Two |  |  |  |  |  |  |  |  |  |
| 9. Arthritis + Diabetes | | | 103 (31.7) | | 325 | | 2.01 | 1.55 | 2.60 |
| 10. Arthritis + Heart disease | | | 97 (37.2) | | 261 | | 2.14 | 1.62 | 2.82 |
| 11. Arthritis + Hypertension | | | 799 (30.4) | | 2628 | | 1.60 | 1.42 | 1.81 |
| 12. Arthritis + Pulmonary disease | | | 49 (38.6) | | 127 | | 2.26 | 1.54 | 3.32 |
| 13. Arthritis + Cancer | | | 24 (30.8) | | 78 | | 1.60 | 0.95 | 2.70 |
| 14. Arthritis + Stroke | | | 46 (54.8) | | 84 | | 4.14 | 2.59 | 6.63 |
| 15. Arthritis + Osteoporosis | | | 252 (33.3) | | 757 | | 1.66 | 1.38 | 1.99 |
| (Continued to next page) | | | | | | | | | |

| Supplementary Material 3. Continued | | | |  | |  | |  | | |  | |  | |
| --- | --- | --- | --- | --- | --- | --- | --- | --- | --- | --- | --- | --- | --- | --- |
|  |  |  |  | |  | |  | |  |  | |  | |  |
| Chronic condition combinations | | | Depression | | | | | | | | | | | |
|  |  |  | Yes | Total | | | | OR | | | 95% CI | | | |
|  |  |  | N (%) | N | | | |  |  |  |  |  |  |  |
| 16. Diabetes + Heart disease | | | 35 (27.1) | 129 | | | | 1.89 | | | 1.24 | | 2.88 | |
| 17. Diabetes + Hypertension | | | 437 (23.9) | 1828 | | | | 1.58 | | | 1.37 | | 1.81 | |
| 18. Diabetes + Pulmonary disease | | | 12 (50.0) | 24 | | | | 4.28 | | | 1.80 | | 10.21 | |
| 19. Diabetes + Cancer | | | 24 (40.7) | 59 | | | | 4.09 | | | 2.33 | | 7.16 | |
| 20. Diabetes + Stroke | | | 30 (52.6) | 57 | | | | 4.95 | | | 2.78 | | 8.81 | |
| 21. Diabetes + Osteoporosis | | | 17 (25.8) | 66 | | | | 1.46 | | | 0.81 | | 2.65 | |
| 22. Heart disease + Hypertension | | | 160 (22.4) | 713 | | | | 1.43 | | | 1.17 | | 1.75 | |
| 23. Heart disease + Pulmonary disease | | | 20 (47.6) | 42 | | | | 3.40 | | | 1.78 | | 6.50 | |
| 24. Heart disease + Cancer | | | 7 (28.0) | 25 | | | | 2.15 | | | 0.83 | | 5.59 | |
| 25. Heart disease + Stroke | | | 11 (36.7) | 30 | | | | 1.82 | | | 0.82 | | 4.03 | |
| 26. Heart disease + Osteoporosis | | | 19 (34.6) | 55 | | | | 1.83 | | | 1.02 | | 3.30 | |
| 27. Hypertension + Pulmonary disease | | | 62 (32.3) | 192 | | | | 2.00 | | | 1.43 | | 2.78 | |
| 28. Hypertension + Cancer | | | 48 (29.5) | 163 | | | | 2.25 | | | 1.56 | | 3.25 | |
| 29. Hypertension + Stroke | | | 195 (38.0) | 513 | | | | 2.53 | | | 2.06 | | 3.12 | |
| 30. Hypertension + Osteoporosis | | | 171 (29.3) | 583 | | | | 1.50 | | | 1.22 | | 1.85 | |
| 31. Pulmonary disease + Cancer | | | 6 (42.9) | 14 | | | | 3.92 | | | 1.28 | | 11.98 | |
| 32. Pulmonary disease + Stroke | | | 10 (66.7) | 15 | | | | 6.78 | | | 2.19 | | 20.99 | |
| 33. Pulmonary disease + Osteoporosis | | | 10 (34.5) | 29 | | | | 2.27 | | | 1.01 | | 5.12 | |
| (Continued to next page) | | | | | | | | | | | | | | |

| Supplementary Material 3. Continued | | | | |  |  |  |  |  |
| --- | --- | --- | --- | --- | --- | --- | --- | --- | --- |
|  |  |  |  |  |  |  |  |  |  |
| Chronic condition combinations | | | Depression | | | | | | |
|  |  |  | Yes | | Total | | OR | 95% CI | |
|  |  |  | N (%) | | N | |  |  |  |
| 34. Cancer + Stroke | | | 5 (55.6) | | 9 | | 4.21 | 0.97 | 18.17 |
| 35. Cancer + Osteoporosis | | | 8 (36.4) | | 22 | | 2.84 | 1.10 | 7.35 |
| 36. Stroke + Osteoporosis | | | 4 (26.7) | | 15 | | 1.14 | 0.33 | 3.90 |
| Three |  |  |  | |  | |  |  |  |
| 37. Arthritis + Diabetes + Heart disease | | | 20 (41.7) | | 48 | | 2.43 | 1.31 | 4.49 |
| 38. Arthritis + Diabetes + Hypertension | | | 353 (36.0) | | 982 | | 2.02 | 1.72 | 2.38 |
| 39. Arthritis + Diabetes + Pulmonary disease | | | 6 (33.3) | | 18 | | 1.77 | 0.64 | 4.91 |
| 40. Arthritis + Diabetes + Cancer | | | 9 (45.0) | | 20 | | 3.16 | 1.24 | 8.05 |
| 41. Arthritis + Diabetes + Stroke | | | 7 (46.7) | | 15 | | 3.39 | 1.11 | 10.36 |
| 42. Arthritis + Diabetes + Osteoporosis | | | 36 (31.3) | | 115 | | 1.40 | 0.91 | 2.15 |
| 43. Arthritis + Heart disease + Hypertension | | | 165 (39.4) | | 419 | | 2.23 | 1.78 | 2.79 |
| 44. Arthritis + Heart disease + Pulmonary disease | | | 7 (58.3) | | 12 | | 2.76 | 0.84 | 9.06 |
| 45. Arthritis + Heart disease + Cancer | | | 3 (42.9) | | 7 | | 2.61 | 0.56 | 12.13 |
| 46. Arthritis + Heart disease + Stroke | | | 7 (50.0) | | 14 | | 2.77 | 0.91 | 8.43 |
| 47. Arthritis + Heart disease + Osteoporosis | | | 34 (49.3) | | 69 | | 2.65 | 1.60 | 4.40 |
| 48. Arthritis + Hypertension + Pulmonary disease | | | 49 (39.2) | | 125 | | 2.09 | 1.41 | 3.08 |
| 49. Arthritis + Hypertension + Cancer | | | 20 (32.8) | | 61 | | 1.82 | 1.03 | 3.24 |
| 50. Arthritis + Hypertension + Stroke | | | 112 (47.5) | | 236 | | 2.69 | 2.02 | 3.58 |
| (Continued to next page) | | | | | | | | | |

| Supplementary Material 3. Continued | | | |  | |  | |  | | |  | |  |  |
| --- | --- | --- | --- | --- | --- | --- | --- | --- | --- | --- | --- | --- | --- | --- |
|  |  |  |  | |  | |  | |  |  | |  | |  |
| Chronic condition combinations | | | Depression | | | | | | | | | | |  |
|  |  |  | Yes | Total | | | | OR | | | 95% CI | | |  |
|  |  |  | N (%) | N | | | |  |  |  |  |  |  |  |
| 51. Arthritis + Hypertension + Osteoporosis | | | 348 (36.7) | 949 | | | | 1.84 | | | 1.57 | | 2.17 |  |
| 52. Arthritis + Pulmonary disease + Cancer | | | 2 (50.0) | 4 | | | | 3.92 | | | 0.54 | | 28.72 |  |
| 53. Arthritis + Pulmonary disease + Stroke | | | 1 (50.0) | 2 | | | | 2.91 | | | 0.14 | | 59.31 |  |
| 54. Arthritis + Pulmonary disease + Osteoporosis | | | 24 (53.3) | 45 | | | | 3.13 | | | 1.64 | | 5.95 |  |
| 55. Arthritis + Cancer + Stroke | | | 0 (0.0) | 1 | | | | - | | | - | | - |  |
| 56. Arthritis + Cancer + Osteoporosis | | | 15 (62.5) | 24 | | | | 7.80 | | | 3.21 | | 18.96 |  |
| 57. Arthritis + Stroke + Osteoporosis | | | 10 (52.6) | 19 | | | | 3.28 | | | 1.23 | | 8.75 |  |
| 58. Diabetes + Heart disease + Hypertension | | | 116 (33.2) | 349 | | | | 2.58 | | | 2.01 | | 3.31 |  |
| 59. Diabetes + Heart disease + Pulmonary disease | | | 5 (50.0) | 10 | | | | 4.09 | | | 1.12 | | 14.98 |  |
| 60. Diabetes + Heart disease + Cancer | | | 2 (33.3) | 6 | | | | 3.29 | | | 0.51 | | 21.25 |  |
| 61. Diabetes + Heart disease + Stroke | | | 3 (33.3) | 9 | | | | 1.55 | | | 0.35 | | 6.88 |  |
| 62. Diabetes + Heart disease + Osteoporosis | | | 5 (45.5) | 11 | | | | 3.37 | | | 0.98 | | 11.58 |  |
| 63. Diabetes + Hypertension + Pulmonary disease | | | 27 (33.8) | 80 | | | | 1.89 | | | 1.14 | | 3.13 |  |
| 64. Diabetes + Hypertension + Cancer | | | 30 (39.5) | 76 | | | | 3.32 | | | 2.01 | | 5.50 |  |
| 65. Diabetes + Hypertension + Stroke | | | 84 (37.5) | 224 | | | | 2.60 | | | 1.93 | | 3.52 |  |
| 66. Diabetes + Hypertension + Osteoporosis | | | 57 (35.6) | 160 | | | | 2.03 | | | 1.43 | | 2.89 |  |
| 67. Diabetes + Pulmonary disease + Cancer | | | 2 (100.0) | 2 | | | | - | | | - | | - |  |
| 68. Diabetes + Pulmonary disease + Stroke | | | 0 (0.0) | 1 | | | | - | | | - | | - |  |
| (Continued to next page) | | | | | | | | | | | | | |  |

| Supplementary Material 3. Continued | | | |  | | |  | |  | |  | |  | |
| --- | --- | --- | --- | --- | --- | --- | --- | --- | --- | --- | --- | --- | --- | --- |
|  |  |  |  | |  |  | |  | |  | |  | |  |
| Chronic condition combinations | | | Depression | | | | | | | | | | | |
|  |  |  | Yes | Total | | | | | OR | | 95% CI | | | |
|  |  |  | N (%) | N | | | | |  |  |  |  |  |  |
| 69. Diabetes + Pulmonary disease + Osteoporosis | | | 1 (33.3) | 3 | | | | | 2.31 | | 0.20 | | 26.76 | |
| 70. Diabetes + Cancer + Stroke | | | - | - | | | | | - | | - | | - | |
| 71. Diabetes + Cancer + Osteoporosis | | | 0 (0.0) | 4 | | | | | - | | - | | - | |
| 72. Diabetes + Stroke + Osteoporosis | | | 3 (100.0) | 3 | | | | | - | | - | | - | |
| 73. Heart disease + Hypertension + Pulmonary disease | | | 12 (28.6) | 42 | | | | | 1.44 | | 0.71 | | 2.93 | |
| 74. Heart disease + Hypertension + Cancer | | | 10 (38.5) | 26 | | | | | 3.18 | | 1.39 | | 7.30 | |
| 75. Heart disease + Hypertension + Stroke | | | 27 (36.5) | 74 | | | | | 2.32 | | 1.39 | | 3.88 | |
| 76. Heart disease + Hypertension + Osteoporosis | | | 27 (29.4) | 92 | | | | | 1.34 | | 0.83 | | 2.17 | |
| 77. Heart disease + Pulmonary disease + Cancer | | | - | - | | | | | - | | - | | - | |
| 78. Heart disease + Pulmonary disease + Stroke | | | 0 (0.0) | 1 | | | | | - | | - | | - | |
| 79. Heart disease + Pulmonary disease + Osteoporosis | | | 2 (66.7) | 3 | | | | | 4.44 | | 0.40 | | 49.39 | |
| 80. Heart disease + Cancer + Stroke | | | 0 (0.0) | 2 | | | | | - | | - | | - | |
| 81. Heart disease + Cancer + Osteoporosis | | | 2 (100.0) | 2 | | | | | - | | - | | - | |
| 82. Heart disease + Stroke + Osteoporosis | | | 2 (100.0) | 2 | | | | | - | | - | | - | |
| 83. Hypertension + Pulmonary disease + Cancer | | | 7 (77.8) | 9 | | | | | 10.77 | | 1.95 | | 59.60 | |
| 84. Hypertension + Pulmonary disease + Stroke | | | 8 (33.3) | 24 | | | | | 2.07 | | 0.83 | | 5.15 | |
| 85. Hypertension + Pulmonary disease + Osteoporosis | | | 8 (38.1) | 21 | | | | | 1.97 | | 0.77 | | 5.03 | |
| 86. Hypertension + Cancer + Stroke | | | 5 (29.4) | 17 | | | | | 1.80 | | 0.59 | | 5.51 | |
| (Continued to next page) | | | | | | | | | | | | | | |

| Supplementary Material 3. Continued | | | | |  |  |  |  |  |
| --- | --- | --- | --- | --- | --- | --- | --- | --- | --- |
|  |  |  |  |  |  |  |  |  |  |
| Chronic condition combinations | | | Depression | | | | | | |
|  |  |  | Yes | | Total | | OR | 95% CI | |
|  |  |  | N (%) | | N | |  |  |  |
| 87. Hypertension + Cancer + Osteoporosis | | | 7 (31.8) | | 22 | | 1.60 | 0.61 | 4.21 |
| 88. Hypertension + Stroke + Osteoporosis | | | 16 (42.1) | | 38 | | 2.69 | 1.35 | 5.36 |
| 89. Pulmonary disease + Cancer + Stroke | | | - | | - | | - | - | - |
| 90. Pulmonary disease + Cancer + Osteoporosis | | | 2 (50.0) | | 4 | | 3.00 | 0.41 | 21.90 |
| 91. Pulmonary disease + Stroke + Osteoporosis | | | 0 (0.0) | | 1 | | - | - | - |
| 92. Cancer + Stroke + Osteoporosis | | | - | | - | | - | - | - |
| Four |  |  |  | |  | |  |  |  |
| 93. Arthritis + Diabetes + Heart disease + Hypertension | | | 90 (45.9) | | 196 | | 2.75 | 2.02 | 3.75 |
| 94. Arthritis + Diabetes + Heart disease + Pulmonary disease | | | 2 (50.0) | | 4 | | 5.02 | 0.66 | 38.27 |
| 95. Arthritis + Diabetes + Heart disease + Cancer | | | 2 (100.0) | | 2 | | - | - | - |
| 96. Arthritis + Diabetes + Heart disease + Stroke | | | 1 (50.0) | | 2 | | 5.21 | 0.32 | 83.99 |
| 97. Arthritis + Diabetes + Heart disease + Osteoporosis | | | 4 (33.3) | | 12 | | 1.41 | 0.40 | 5.03 |
| 98. Arthritis + Diabetes + Hypertension + Pulmonary disease | | | 19 (51.4) | | 37 | | 3.04 | 1.52 | 6.10 |
| 99. Arthritis + Diabetes + Hypertension + Cancer | | | 15 (38.5) | | 39 | | 2.47 | 1.23 | 4.95 |
| 100. Arthritis + Diabetes + Hypertension + Stroke | | | 73 (57.9) | | 126 | | 4.75 | 3.22 | 7.00 |
| 101. Arthritis + Diabetes + Hypertension + Osteoporosis | | | 143 (42.6) | | 336 | | 2.28 | 1.78 | 2.91 |
| 102. Arthritis + Diabetes + Pulmonary disease + Cancer | | | - | | - | | - | - | - |
| 103. Arthritis + Diabetes + Pulmonary disease + Stroke | | | 1 (100.0) | | 1 | | - | - | - |
| (Continued to next page) | | | | | | | | | |

| Supplementary Material 3. Continued | | | |  | |  | |  | | |  | |  |  |
| --- | --- | --- | --- | --- | --- | --- | --- | --- | --- | --- | --- | --- | --- | --- |
|  |  |  |  | |  | |  | |  |  | |  | |  |
| Chronic condition combinations | | | Depression | | | | | | | | | | |  |
|  |  |  | Yes | Total | | | | OR | | | 95% CI | | |  |
|  |  |  | N (%) | N | | | |  |  |  |  |  |  |  |
| 104. Arthritis + Diabetes + Pulmonary disease + Osteoporosis | | | 2 (66.7) | 3 | | | | 5.29 | | | 0.44 | | 64.23 |  |
| 105. Arthritis + Diabetes + Cancer + Stroke | | | 1 (50.0) | 2 | | | | 2.84 | | | 0.14 | | 56.40 |  |
| 106. Arthritis + Diabetes + Cancer + Osteoporosis | | | 0 (0.0) | 1 | | | | - | | | - | | - |  |
| 107. Arthritis + Diabetes + Stroke + Osteoporosis | | | 5 (83.3) | 6 | | | | 18.45 | | | 1.98 | | 171.78 |  |
| 108. Arthritis + Heart disease + Hypertension + Pulmonary disease | | | 22 (61.1) | 36 | | | | 4.07 | | | 2.00 | | 8.30 |  |
| 109. Arthritis + Heart disease + Hypertension + Cancer | | | 3 (50.0) | 6 | | | | 4.92 | | | 0.96 | | 25.24 |  |
| 110. Arthritis + Heart disease + Hypertension + Stroke | | | 29 (58.0) | 50 | | | | 4.53 | | | 2.48 | | 8.29 |  |
| 111. Arthritis + Heart disease + Hypertension + Osteoporosis | | | 56 (38.6) | 145 | | | | 1.94 | | | 1.35 | | 2.79 |  |
| 112. Arthritis + Heart disease + Pulmonary disease + Cancer | | | - | - | | | | - | | | - | | - |  |
| 113. Arthritis + Heart disease + Pulmonary disease + Stroke | | | - | - | | | | - | | | - | | - |  |
| 114. Arthritis + Heart disease + Pulmonary disease + Osteoporosis | | | 4 (50.0) | 8 | | | | 1.74 | | | 0.41 | | 7.35 |  |
| 115. Arthritis + Heart disease + Cancer + Stroke | | | - | - | | | | - | | | - | | - |  |
| 116. Arthritis + Heart disease + Cancer + Osteoporosis | | | 4 (100.0) | 4 | | | | - | | | - | | - |  |
| 117. Arthritis + Heart disease + Stroke + Osteoporosis | | | 1 (100.0) | 1 | | | | - | | | - | | - |  |
| 118. Arthritis + Hypertension + Pulmonary disease + Cancer | | | 3 (75.0) | 4 | | | | 4.67 | | | 0.44 | | 49.35 |  |
| 119. Arthritis + Hypertension + Pulmonary disease + Stroke | | | 10 (76.9) | 13 | | | | 9.20 | | | 2.33 | | 36.29 |  |
| 120. Arthritis + Hypertension + Pulmonary disease + Osteoporosis | | | 32 (50.8) | 63 | | | | 2.70 | | | 1.58 | | 4.61 |  |
| 121. Arthritis + Hypertension + Cancer + Stroke | | | 4 (44.4) | 9 | | | | 2.51 | | | 0.61 | | 10.40 |  |
| (Continued to next page) | | | | | | | | | | | | | |  |

| Supplementary Material 3. Continued | | | |  | |  | |  | | |  | |  |  |
| --- | --- | --- | --- | --- | --- | --- | --- | --- | --- | --- | --- | --- | --- | --- |
|  |  |  |  | |  | |  | |  |  | |  | |  |
| Chronic condition combinations | | | Depression | | | | | | | | | | |  |
|  |  |  | Yes | Total | | | | OR | | | 95% CI | | |  |
|  |  |  | N (%) | N | | | |  |  |  |  |  |  |  |
| 122. Arthritis + Hypertension + Cancer + Osteoporosis | | | 5 (17.9) | 28 | | | | 0.77 | | | 0.28 | | 2.10 |  |
| 123. Arthritis + Hypertension + Stroke + Osteoporosis | | | 56 (56.0) | 100 | | | | 3.68 | | | 2.40 | | 5.65 |  |
| 124. Arthritis + Pulmonary disease + Cancer + Stroke | | | - | - | | | | - | | | - | | - |  |
| 125. Arthritis + Pulmonary disease + Cancer + Osteoporosis | | | 1 (33.3) | 3 | | | | 1.38 | | | 0.09 | | 20.54 |  |
| 126. Arthritis + Pulmonary disease + Stroke + Osteoporosis | | | 1 (33.3) | 3 | | | | 1.81 | | | 0.13 | | 25.05 |  |
| 127. Arthritis + Cancer + Stroke + Osteoporosis | | | - | - | | | | - | | | - | | - |  |
| 128. Diabetes + Heart disease + Hypertension + Pulmonary disease | | | 12 (48.0) | 25 | | | | 3.55 | | | 1.55 | | 80.10 |  |
| 129. Diabetes + Heart disease + Hypertension + Cancer | | | 5 (33.3) | 15 | | | | 2.97 | | | 0.97 | | 9.10 |  |
| 130. Diabetes + Heart disease + Hypertension + Stroke | | | 31 (53.5) | 58 | | | | 5.34 | | | 3.03 | | 9.39 |  |
| 131. Diabetes + Heart disease + Hypertension + Osteoporosis | | | 23 (54.8) | 42 | | | | 5.13 | | | 2.69 | | 9.76 |  |
| 132. Diabetes + Heart disease + Pulmonary disease + Cancer | | | - | - | | | | - | | | - | | - |  |
| 133. Diabetes + Heart disease + Pulmonary disease + Stroke | | | 0 (0.0) | 1 | | | | - | | | - | | - |  |
| 134. Diabetes + Heart disease + Pulmonary disease + Osteoporosis | | | - | - | | | | - | | | - | | - |  |
| 135. Diabetes + Heart disease + Cancer + Stroke | | | 1 (100.0) | 1 | | | | - | | | - | | - |  |
| 136. Diabetes + Heart disease + Cancer + Osteoporosis | | | 0 (0.0) | 1 | | | | - | | | - | | - |  |
| 137. Diabetes + Heart disease + Stroke + Osteoporosis | | | - | - | | | | - | | | - | | - |  |
| 138. Diabetes + Hypertension + Pulmonary disease + Cancer | | | 2 (66.7) | 3 | | | | 17.37 | | | 1.40 | | 215.12 |  |
| 139. Diabetes + Hypertension + Pulmonary disease + Stroke | | | 4 (36.4) | 11 | | | | 1.54 | | | 0.42 | | 5.67 |  |
| 140. Diabetes + Hypertension + Pulmonary disease + Osteoporosis | | | 1 (20.0) | 5 | | | | 0.89 | | | 0.08 | | 9.70 |  |
| (Continued to next page) | | | | | | | | | | | | | |  |

| Supplementary Material 3. Continued | | |  |  |  |  |  |  |  |
| --- | --- | --- | --- | --- | --- | --- | --- | --- | --- |
|  |  |  |  |  |  |  |  |  |  |
| Chronic condition combinations | | | Depression | | | | | | |
|  |  |  | Yes | | Total | | OR | 95% CI | |
|  |  |  | N (%) | | N | |  |  |  |
| 141. Diabetes + Hypertension + Cancer + Stroke | | | 3 (37.5) | | 8 | | 3.66 | 0.84 | 15.92 |
| 142. Diabetes + Hypertension + Cancer + Osteoporosis | | | 1 (20.0) | | 5 | | 0.96 | 0.10 | 8.83 |
| 143. Diabetes + Hypertension + Stroke + Osteoporosis | | | 3 (25.0) | | 12 | | 1.26 | 0.33 | 4.92 |
| 144. Diabetes + Pulmonary disease + Cancer + Stroke | | | - | | - | | - | - | - |
| 145. Diabetes + Pulmonary disease + Cancer + Osteoporosis | | | 1 (50.0) | | 2 | | 4.12 | 0.25 | 67.67 |
| 146. Diabetes + Pulmonary disease + Stroke + Osteoporosis | | | - | | - | | - | - | - |
| 147. Diabetes + Cancer + Stroke + Osteoporosis | | | - | | - | | - | - | - |
| 148. Heart disease + Hypertension + Pulmonary disease + Cancer | | | - | | - | | - | - | - |
| 149. Heart disease + Hypertension + Pulmonary disease + Stroke | | | 3 (42.9) | | 7 | | 6.23 | 1.36 | 28.55 |
| 150. Heart disease + Hypertension + Pulmonary disease + Osteoporosis | | | 2 (33.3) | | 6 | | 2.12 | 0.34 | 13.15 |
| 151. Heart disease + Hypertension + Cancer + Stroke | | | 3 (75.0) | | 4 | | 7.43 | 0.76 | 72.65 |
| 152. Heart disease + Hypertension + Cancer + Osteoporosis | | | 1 (33.3) | | 3 | | 1.32 | 0.11 | 16.40 |
| 153. Heart disease + Hypertension + Stroke + Osteoporosis | | | 4 (80.0) | | 5 | | 8.15 | 0.86 | 76.94 |
| 154. Heart disease + Pulmonary disease + Cancer + Stroke | | | 0 (0.0) | | 1 | | - | - | - |
| 155. Heart disease + Pulmonary disease + Cancer + Osteoporosis | | | - | | - | | - | - | - |
| 156. Heart disease + Pulmonary disease + Stroke + Osteoporosis | | | - | | - | | - | - | - |
| 157. Heart disease + Cancer + Stroke + Osteoporosis | | | - | | - | | - | - | - |
| 158. Hypertension + Pulmonary disease + Cancer + Stroke | | | 1 (50.0) | | 2 | | 2.50 | 0.07 | 94.51 |
| (Continued to next page) | | | | | | | | | |

| Supplementary Material 3. Continued | | | | |  |  |  |  |  |
| --- | --- | --- | --- | --- | --- | --- | --- | --- | --- |
|  |  |  |  |  |  |  |  |  |  |
| Chronic condition combinations | | | Depression | | | | | | |
|  |  |  | Yes | | Total | | OR | 95% CI | |
|  |  |  | N (%) | | N | |  |  |  |
| 159. Hypertension + Pulmonary disease + Cancer + Osteoporosis | | | 1 (50.0) | | 2 | | 1.96 | 0.10 | 36.91 |
| 160. Hypertension + Pulmonary disease + Stroke + Osteoporosis | | | 1 (100.0) | | 1 | | - | - | - |
| 161. Hypertension + Cancer + Stroke + Osteoporosis | | | 2 (66.7) | | 3 | | 6.01 | 0.26 | 138.69 |
| 162. Pulmonary disease + Cancer + Stroke + Osteoporosis | | | - | | - | | - | - | - |
| Five |  |  |  | |  | |  |  |  |
| 163. Arthritis + Diabetes + Heart disease + Hypertension + Pulmonary disease | | | 11 (61.1) | | 18 | | 4.94 | 1.76 | 13.85 |
| 164. Arthritis + Diabetes + Heart disease + Hypertension + Cancer | | | 4 (80.0) | | 5 | | 21.74 | 2.00 | 236.57 |
| 165. Arthritis + Diabetes + Heart disease + Hypertension + Stroke | | | 17 (68.0) | | 25 | | 5.48 | 2.21 | 13.54 |
| 166. Arthritis + Diabetes + Heart disease + Hypertension + Osteoporosis | | | 43 (53.8) | | 80 | | 3.12 | 1.94 | 5.02 |
| 167. Arthritis + Diabetes + Heart disease + Pulmonary disease + Cancer | | | - | | - | | - | - | - |
| 168. Arthritis + Diabetes + Heart disease + Pulmonary disease + Stroke | | | 0 (0.0) | | 1 | | - | - | - |
| 169. Arthritis + Diabetes + Heart disease + Pulmonary disease + Osteoporosis | | | 0 (0.0) | | 1 | | - | - | - |
| 170. Arthritis + Diabetes + Heart disease + Cancer + Stroke | | | 0 (0.0) | | 1 | | - | - | - |
| 171. Arthritis + Diabetes + Heart disease + Cancer + Osteoporosis | | | 0 (0.0) | | 2 | | - | - | - |
| 172. Arthritis + Diabetes + Heart disease + Stroke + Osteoporosis | | | 1 (100.0) | | 1 | | - | - | - |
| 173. Arthritis + Diabetes + Hypertension + Pulmonary disease + Cancer | | | 0 (0.0) | | 1 | | - | - | - |
| 174. Arthritis + Diabetes + Hypertension + Pulmonary disease + Stroke | | | 4 (57.1) | | 7 | | 4.31 | 0.94 | 19.71 |
| 175. Arthritis + Diabetes + Hypertension + Pulmonary disease + Osteoporosis | | | 15 (65.2) | | 23 | | 3.69 | 1.49 | 9.16 |
| (Continued to next page) | | | | | | | | | |

| Supplementary Material 3. Continued | | |  |  |  |  |  |  |  |
| --- | --- | --- | --- | --- | --- | --- | --- | --- | --- |
|  |  |  |  |  |  |  |  |  |  |
| Chronic condition combinations | | | Depression | | | | | | |
|  |  |  | Yes | | Total | | OR | 95% CI | |
|  |  |  | N (%) | | N | |  |  |  |
| 176. Arthritis + Diabetes + Hypertension + Cancer + Stroke | | | 2 (100.0) | | 2 | | - | - | - |
| 177. Arthritis + Diabetes + Hypertension + Cancer + Osteoporosis | | | 8 (57.1) | | 14 | | 2.93 | 0.94 | 9.11 |
| 178. Arthritis + Diabetes + Hypertension + Stroke + Osteoporosis | | | 19 (52.8) | | 36 | | 3.02 | 1.50 | 6.08 |
| 179. Arthritis + Diabetes + Pulmonary disease + Cancer + Stroke | | | - | | - | | - | - | - |
| 180. Arthritis + Diabetes + Pulmonary disease + Cancer + Osteoporosis | | | - | | - | | - | - | - |
| 181. Arthritis + Diabetes + Pulmonary disease + Stroke + Osteoporosis | | | - | | - | | - | - | - |
| 182. Arthritis + Diabetes + Cancer + Stroke + Osteoporosis | | | - | | - | | - | - | - |
| 183. Arthritis + Heart disease + Hypertension + Pulmonary disease + Cancer | | | - | | - | | - | - | - |
| 184. Arthritis + Heart disease + Hypertension + Pulmonary disease + Stroke | | | 1 (33.3) | | 3 | | 1.13 | 0.09 | 14.06 |
| 185. Arthritis + Heart disease + Hypertension + Pulmonary disease + Osteoporosis | | | 9 (52.9) | | 17 | | 3.38 | 1.25 | 9.12 |
| 186. Arthritis + Heart disease + Hypertension + Cancer + Stroke | | | 2 (66.7) | | 3 | | 12.13 | 1.03 | 143.07 |
| 187. Arthritis + Heart disease + Hypertension + Cancer + Osteoporosis | | | 4 (80.0) | | 5 | | 9.92 | 1.10 | 89.48 |
| 188. Arthritis + Heart disease + Hypertension + Stroke + Osteoporosis | | | 12 (63.2) | | 19 | | 3.73 | 1.37 | 10.15 |
| 189. Arthritis + Heart disease + Pulmonary disease + Cancer + Stroke | | | - | | - | | - | - | - |
| 190. Arthritis + Heart disease + Pulmonary disease + Cancer + Osteoporosis | | | - | | - | | - | - | - |
| 191. Arthritis + Heart disease + Pulmonary disease + Stroke + Osteoporosis | | | 3 (75.0) | | 4 | | 9.84 | 1.00 | 96.88 |
| 192. Arthritis + Heart disease + Cancer + Stroke + Osteoporosis | | | 1 (100.0) | | 1 | | - | - | - |
| 193. Arthritis + Hypertension + Pulmonary disease + Cancer + Stroke | | | 0 (0.0) | | 1 | | - | - | - |
| (Continued to next page) | | | | | | | | | |

| Supplementary Material 3. Continued | | |  |  |  |  |  |  |  |
| --- | --- | --- | --- | --- | --- | --- | --- | --- | --- |
|  |  |  |  |  |  |  |  |  |  |
| Chronic condition combinations | | | Depression | | | | | | |
|  |  |  | Yes | | Total | | OR | 95% CI | |
|  |  |  | N (%) | | N | |  |  |  |
| 194. Arthritis + Hypertension + Pulmonary disease + Cancer + Osteoporosis | | | 1 (100.0) | | 1 | | - | - | - |
| 195. Arthritis + Hypertension + Pulmonary disease + Stroke + Osteoporosis | | | 5 (62.5) | | 8 | | 3.74 | 0.81 | 17.27 |
| 196. Arthritis + Hypertension + Cancer + Stroke + Osteoporosis | | | 1 (100.0) | | 1 | | - | - | - |
| 197. Arthritis + Pulmonary disease + Cancer + Stroke + Osteoporosis | | | - | | - | | - | - | - |
| 198. Diabetes + Heart disease + Hypertension + Pulmonary disease + Cancer | | | 0 (0.0) | | 1 | | - | - | - |
| 199. Diabetes + Heart disease + Hypertension + Pulmonary disease + Stroke | | | 3 (60.0) | | 5 | | 8.79 | 1.28 | 60.39 |
| 200. Diabetes + Heart disease + Hypertension + Pulmonary disease + Osteoporosis | | | 2 (33.3) | | 6 | | 3.17 | 0.56 | 17.77 |
| 201. Diabetes + Heart disease + Hypertension + Cancer + Stroke | | | 1 (100.0) | | 1 | | - | - | - |
| 202. Diabetes + Heart disease + Hypertension + Cancer + Osteoporosis | | | 0 (0.0) | | 1 | | - | - | - |
| 203. Diabetes + Heart disease + Hypertension + Stroke + Osteoporosis | | | 2 (100.0) | | 2 | | - | - | - |
| 204. Diabetes + Heart disease + Pulmonary disease + Cancer + Stroke | | | - | | - | | - | - | - |
| 205. Diabetes + Heart disease + Pulmonary disease + Cancer + Osteoporosis | | | - | | - | | - | - | - |
| 206. Diabetes + Heart disease + Pulmonary disease + Stroke + Osteoporosis | | | - | | - | | - | - | - |
| 207. Diabetes + Heart disease + Cancer + Stroke + Osteoporosis | | | - | | - | | - | - | - |
| 208. Diabetes + Hypertension + Pulmonary disease + Cancer + Stroke | | | 0 (0.0) | | 2 | | - | - | - |
| 209. Diabetes + Hypertension + Pulmonary disease + Cancer + Osteoporosis | | | - | | - | | - | - | - |
| 210. Diabetes + Hypertension + Pulmonary disease + Stroke + Osteoporosis | | | 1 (100.0) | | 1 | | - | - | - |
| 211. Diabetes + Hypertension + Cancer + Stroke + Osteoporosis | | | - | | - | | - | - | - |
| (Continued to next page) | | | | | | | | | |

| Supplementary Material 3. Continued | | |  |  |  |  |  |  |  |
| --- | --- | --- | --- | --- | --- | --- | --- | --- | --- |
|  |  |  |  |  |  |  |  |  |  |
| Chronic condition combinations | | | Depression | | | | | | |
|  |  |  | Yes | | Total | | OR | 95% CI | |
|  |  |  | N (%) | | N | |  |  |  |
| 212. Diabetes + Pulmonary disease + Cancer + Stroke + Osteoporosis | | | - | | - | | - | - | - |
| 213. Heart disease + Hypertension + Pulmonary disease + Cancer + Stroke | | | - | | - | | - | - | - |
| 214. Heart disease + Hypertension + Pulmonary disease + Cancer + Osteoporosis | | | - | | - | | - | - | - |
| 215. Heart disease + Hypertension + Pulmonary disease + Stroke + Osteoporosis | | | 1 (100.0) | | 1 | | - | - | - |
| 216. Heart disease + Hypertension + Cancer + Stroke + Osteoporosis | | | - | | - | | - | - | - |
| 217. Heart disease + Pulmonary disease + Cancer + Stroke + Osteoporosis | | | - | | - | | - | - | - |
| 218. Hypertension + Pulmonary disease + Cancer + Stroke + Osteoporosis | | | - | | - | | - | - | - |
| Six |  |  |  | |  | |  |  |  |
| 219. Arthritis + Diabetes + Heart disease + Hypertension + Pulmonary disease + Cancer | | | - | | - | | - | - | - |
| 220. Arthritis + Diabetes + Heart disease + Hypertension + Pulmonary disease + Stroke | | | - | | - | | - | - | - |
| 221. Arthritis + Diabetes + Heart disease + Hypertension + Pulmonary disease + Osteoporosis | | | 8 (80.0) | | 10 | | 6.90 | 1.38 | 34.62 |
| 222. Arthritis + Diabetes + Heart disease + Hypertension + Cancer + Stroke | | | - | | - | | - | - | - |
| 223. Arthritis + Diabetes + Heart disease + Hypertension + Cancer + Osteoporosis | | | 0 (0.0) | | 2 | | - | - | - |
| 224. Arthritis + Diabetes + Heart disease + Hypertension + Stroke + Osteoporosis | | | 10 (52.6) | | 19 | | 2.28 | 0.85 | 6.09 |
| 225. Arthritis + Diabetes + Heart disease + Pulmonary disease + Cancer + Stroke | | | - | | - | | - | - | - |
| 226. Arthritis + Diabetes + Heart disease + Pulmonary disease + Cancer + Osteoporosis | | | - | | - | | - | - | - |
| 227. Arthritis + Diabetes + Heart disease + Pulmonary disease + Stroke + Osteoporosis | | | - | | - | | - | - | - |
| 228. Arthritis + Diabetes + Heart disease + Cancer + Stroke + Osteoporosis | | | - | | - | | - | - | - |
| (Continued to next page) | | | | | | | | | |

| Supplementary Material 3. Continued | | |  |  |  |  |  |  |  | |
| --- | --- | --- | --- | --- | --- | --- | --- | --- | --- | --- |
|  |  |  |  |  |  |  |  |  |  | |
| Chronic condition combinations | | | Depression | | | | | | |  |
|  |  |  | Yes | | Total | | OR | 95% CI | | |
|  |  |  | N (%) | | N | |  |  |  |  |
| 229. Arthritis + Diabetes + Hypertension + Pulmonary disease + Cancer + Stroke | | | - | | - | | - | - | - | |
| 230. Arthritis + Diabetes + Hypertension + Pulmonary disease + Cancer + Osteoporosis | | | 1 (100.0) | | 1 | | - | - | - | |
| 231. Arthritis + Diabetes + Hypertension + Pulmonary disease + Stroke + Osteoporosis | | | 1 (50.0) | | 2 | | 2.56 | 0.16 | 41.14 | |
| 232. Arthritis + Diabetes + Hypertension + Cancer + Stroke + Osteoporosis | | | - | | - | | - | - | - | |
| 233. Arthritis + Diabetes + Pulmonary disease + Cancer + Stroke + Osteoporosis | | | - | | - | | - | - | - | |
| 234. Arthritis + Heart disease + Hypertension + Pulmonary disease + Cancer + Stroke | | | - | | - | | - | - | - | |
| 235. Arthritis + Heart disease + Hypertension + Pulmonary disease + Cancer + Osteoporosis | | | - | | - | | - | - | - | |
| 236. Arthritis + Heart disease + Hypertension + Pulmonary disease + Stroke + Osteoporosis | | | 1 (50.0) | | 2 | | 5.53 | 0.30 | 103.61 | |
| 237. Arthritis + Heart disease + Hypertension + Cancer + Stroke + Osteoporosis | | | - | | - | | - | - | - | |
| 238. Arthritis + Heart disease + Pulmonary disease + Cancer + Stroke + Osteoporosis | | | - | | - | | - | - | - | |
| 239. Arthritis + Hypertension + Pulmonary disease + Cancer + Stroke + Osteoporosis | | | - | | - | | - | - | - | |
| 240. Diabetes + Heart disease + Hypertension + Pulmonary disease + Cancer + Stroke | | | 0 (0.0) | | 1 | | - | - | - | |
| 241. Diabetes + Heart disease + Hypertension + Pulmonary disease + Cancer + Osteoporosis | | | - | | - | | - | - | - | |
| 242. Diabetes + Heart disease + Hypertension + Pulmonary disease + Stroke + Osteoporosis | | | 0 (0.0) | | 1 | | - | - | - | |
| 243. Diabetes + Heart disease + Hypertension + Cancer + Stroke + Osteoporosis | | | - | | - | | - | - | - | |
| 244. Diabetes + Heart disease + Pulmonary disease + Cancer + Stroke + Osteoporosis | | | - | | - | | - | - | - | |
| 245. Diabetes + Hypertension + Pulmonary disease + Cancer + Stroke + Osteoporosis | | | - | | - | | - | - | - | |
| 246. Heart disease + Hypertension + Pulmonary disease + Cancer + Stroke + Osteoporosis | | | - | | - | | - | - | - | |
| (Continued to next page) | | | | | | | | | |  |

| Supplementary Material 3. Continued |  | |  | |  | |  |  | | |  | |  | | |  |
| --- | --- | --- | --- | --- | --- | --- | --- | --- | --- | --- | --- | --- | --- | --- | --- | --- |
|  | |  | |  |  |  | | |  |  | |  | |  |  | |
| Chronic condition combinations | Depression | | | | | | | | | | | | | | |  |
|  | Yes | | | | Total | | | OR | | | 95% CI | | | | |  |
|  | N (%) | | | | N | | |  |  |  |  |  |  |  |  |  |
| Seven | |  | |  |  | | | |  | | |  | |  |  | |
| 247. Arthritis + Diabetes + Heart disease + Hypertension + Pulmonary disease + Cancer + Stroke | - | | | | - | | | - | | | - | | - | | |  |
| 248. Arthritis + Diabetes + Heart disease + Hypertension + Pulmonary disease + Cancer + Osteoporosis | 0 (0.0) | | | | 1 | | | - | | | - | | - | | |  |
| 249. Arthritis + Diabetes + Heart disease + Hypertension + Pulmonary disease + Stroke + Osteoporosis | 2 (66.7) | | | | 3 | | | 2.06 | | | 0.13 | | 32.98 | | |  |
| 250. Arthritis + Diabetes + Heart disease + Hypertension + Cancer + Stroke + Osteoporosis | - | | | | - | | | - | | | - | | - | | |  |
| 251. Arthritis + Diabetes + Heart disease + Pulmonary disease + Cancer + Stroke + Osteoporosis | - | | | | - | | | - | | | - | | - | | |  |
| 252. Arthritis + Diabetes + Hypertension + Pulmonary disease + Cancer + Stroke + Osteoporosis | - | | | | - | | | - | | | - | | - | | |  |
| 253. Arthritis + Heart disease + Hypertension + Pulmonary disease + Cancer + Stroke + Osteoporosis | - | | | | - | | | - | | | - | | - | | |  |
| 254. Diabetes + Heart disease + Hypertension + Pulmonary disease + Cancer + Stroke + Osteoporosis | - | | | | - | | | - | | | - | | - | | |  |
| Eight | |  | |  |  | | | |  | | |  | |  |  | |
| 255. Arthritis + Diabetes + Heart disease + Hypertension + Pulmonary disease + Cancer + Stroke + Osteoporosis | - | | | | - | | | - | | | - | | - | | |  |
| Adjusted for gender, age, marital status, living arrangement, education, type of insurance, current smoking, lack of exercise, high-risk alcohol drinking, restriction on activities of daily living, frequency of contact with people, other chronic conditions, and year. | | | | | | | | | | | | | | | |  |
|  |  |  |  |  |  |  |  |  |  |  |  |  |  |  |  |  |
