# Supplementary material for: Gender differences in the association between multimorbidity and depression in older Korean adults: an analysis of data from the National Survey of Older Koreans (2011-2017)
Source: Epidemiol Health. 2022 May 24;44:e2022049. doi: 10.4178/epih.e2022049 (PMC9714839; doi:10.4178/epih.e2022049)
Supplement: Supplementary Material 4. — The association between chronic conditions combinations and depression by gender [file epih-44-e2022049-suppl4.docx]

| Supplementary Material 4. The association between chronic conditions combinations and depression by gender | | | | | | | | | | | | | | | |  | |  | |  | |  | |  | |  |
| --- | --- | --- | --- | --- | --- | --- | --- | --- | --- | --- | --- | --- | --- | --- | --- | --- | --- | --- | --- | --- | --- | --- | --- | --- | --- | --- |
|  |  |  |  |  |  |  | |  | |  | |  | |  |  | |  | |  | |  | |  | |  | |
| Chronic condition combinations | | | Men | | | | | | | | | | Women | | | | | | | | | | | | |  |
|  |  |  | Depression | | | | | | | | | | Depression | | | | | | | | | | | | |  |
|  |  |  | Yes | | Total | | OR | | 95% CI | | | | Yes | | | Total | | | | OR | | 95% CI | | | |  |
|  |  |  | N (%) | | N | |  |  |  |  |  |  | N (%) | | | N | | | |  |  |  |  |  |  |  |
| Zero | | | 454 (13.9) | | 3259 | | 1.00 | |  | | | | 406 (17.9) | | | 2264 | | | | 1.00 | |  | | | |  |
| One |  |  |  |  |  |  | |  | |  | |  | |  |  | |  | |  | |  | |  | |  | |
| 1. Arthritis | | | 191 (27.7) | | 689 | | 1.61 | | 1.30 | | 1.98 | | 459 (29.7) | | | 1547 | | | | 1.49 | | 1.27 | | 1.75 | |  |
| 2. Diabetes | | | 60 (15.0) | | 401 | | 1.16 | | 0.85 | | 1.58 | | 83 (26.4) | | | 315 | | | | 1.67 | | 1.25 | | 2.24 | |  |
| 3. Heart disease | | | 56 (18.0) | | 312 | | 1.14 | | 0.83 | | 1.58 | | 60 (30.2) | | | 199 | | | | 1.74 | | 1.24 | | 2.46 | |  |
| 4. Hypertension | | | 332 (13.7) | | 2417 | | 1.00 | | 0.85 | | 1.17 | | 461 (20.4) | | | 2261 | | | | 1.06 | | 0.91 | | 1.24 | |  |
| 5. Pulmonary disease | | | 69 (30.9) | | 223 | | 1.94 | | 1.41 | | 2.68 | | 23 (27.1) | | | 85 | | | | 1.30 | | 0.78 | | 2.18 | |  |
| 6. Cancer | | | 67 (31.0) | | 216 | | 2.88 | | 2.08 | | 3.98 | | 38 (44.7) | | | 85 | | | | 3.69 | | 2.30 | | 5.90 | |  |
| 7. Stroke | | | 52 (36.6) | | 142 | | 2.87 | | 1.95 | | 4.20 | | 27 (42.2) | | | 64 | | | | 2.71 | | 1.59 | | 4.62 | |  |
| 8. Osteoporosis | | | 20 (42.6) | | 47 | | 2.53 | | 1.34 | | 4.77 | | 91 (19.7) | | | 461 | | | | 1.02 | | 0.78 | | 1.34 | |  |
| Two |  |  |  |  |  |  | |  | |  | |  | |  |  | |  | |  | |  | |  | |  | |
| 9. Arthritis + Diabetes | | | 29 (31.5) | | 92 | | 2.35 | | 1.45 | | 3.81 | | 74 (31.8) | | | 233 | | | | 1.86 | | 1.36 | | 2.53 | |  |
| 10. Arthritis + Heart disease | | | 30 (38.5) | | 78 | | 2.68 | | 1.62 | | 4.43 | | 67 (36.6) | | | 183 | | | | 1.92 | | 1.37 | | 2.68 | |  |
| 11. Arthritis + Hypertension | | | 151 (28.8) | | 585 | | 1.51 | | 1.20 | | 1.89 | | 648 (31.7) | | | 2043 | | | | 1.59 | | 1.37 | | 1.85 | |  |
| 12. Arthritis + Pulmonary disease | | | 23 (41.8) | | 55 | | 2.93 | | 1.65 | | 5.20 | | 26 (36.1) | | | 72 | | | | 1.80 | | 1.07 | | 3.03 | |  |
| 13. Arthritis + Cancer | | | 12 (36.4) | | 33 | | 2.09 | | 0.96 | | 4.52 | | 12 (26.7) | | | 45 | | | | 1.20 | | 0.59 | | 2.47 | |  |
| 14. Arthritis + Stroke | | | 11 (47.8) | | 23 | | 3.11 | | 1.24 | | 7.78 | | 35 (57.4) | | | 61 | | | | 4.43 | | 2.55 | | 7.71 | |  |
| 15. Arthritis + Osteoporosis | | | 16 (35.6) | | 45 | | 1.80 | | 0.91 | | 3.56 | | 236 (33.2) | | | 712 | | | | 1.60 | | 1.31 | | 1.95 | |  |
| (Continued to next page) | | | | | | | | | | | | | | | | | | | | | | | | | |  |

| Supplementary Material 4. Continued | | |  |  |  |  |  |  |  |  |  |  |  |  |  |  |
| --- | --- | --- | --- | --- | --- | --- | --- | --- | --- | --- | --- | --- | --- | --- | --- | --- |
|  |  |  |  |  |  |  |  |  |  |  |  |  |  |  |  |  |
| Chronic condition combinations | | | Men | | | | | | | Women | | | | | | |
|  |  |  | Depression | | | | | | | Depression | | | | | | |
|  |  |  | Yes | | Total | | OR | 95% CI | | Yes | | Total | | OR | 95% CI | |
|  |  |  | N (%) | | N | |  |  |  | N (%) | | N | |  |  |  |
| 16. Diabetes + Heart disease | | | 15 (21.4) | | 70 | | 1.68 | 0.91 | 3.10 | 20 (33.9) | | 59 | | 2.07 | 1.15 | 3.73 |
| 17. Diabetes + Hypertension | | | 195 (21.2) | | 921 | | 1.57 | 1.28 | 1.92 | 242 (26.7) | | 907 | | 1.55 | 1.28 | 1.88 |
| 18. Diabetes + Pulmonary disease | | | 10 (55.6) | | 18 | | 6.02 | 2.19 | 16.58 | 2 (33.3) | | 6 | | 1.45 | 0.24 | 8.86 |
| 19. Diabetes + Cancer | | | 17 (38.6) | | 44 | | 3.76 | 1.92 | 7.38 | 7 (46.7) | | 15 | | 4.66 | 1.62 | 13.37 |
| 20. Diabetes + Stroke | | | 20 (54.1) | | 37 | | 5.60 | 2.72 | 11.51 | 10 (50.0) | | 20 | | 3.64 | 1.38 | 9.65 |
| 21. Diabetes + Osteoporosis | | | 0 (0.0) | | 3 | | - | - | - | 17 (27.0) | | 63 | | 1.48 | 0.81 | 2.70 |
| 22. Heart disease + Hypertension | | | 86 (22.8) | | 377 | | 1.81 | 1.37 | 2.39 | 74 (22.0) | | 336 | | 1.11 | 0.82 | 1.48 |
| 23. Heart disease + Pulmonary disease | | | 16 (45.7) | | 35 | | 3.11 | 1.52 | 6.36 | 4 (57.1) | | 7 | | 5.10 | 1.00 | 26.01 |
| 24. Heart disease + Cancer | | | 6 (35.3) | | 17 | | 3.27 | 1.05 | 10.02 | 1 (12.5) | | 8 | | 0.71 | 0.08 | 6.07 |
| 25. Heart disease + Stroke | | | 8 (42.1) | | 19 | | 2.61 | 0.95 | 7.13 | 3 (27.3) | | 11 | | 0.90 | 0.23 | 3.52 |
| 26. Heart disease + Osteoporosis | | | 3 (42.9) | | 7 | | 3.28 | 0.70 | 15.32 | 16 (33.3) | | 48 | | 1.63 | 0.86 | 3.08 |
| 27. Hypertension + Pulmonary disease | | | 33 (28.0) | | 118 | | 1.80 | 1.16 | 2.81 | 29 (39.2) | | 74 | | 2.24 | 1.35 | 3.72 |
| 28. Hypertension + Cancer | | | 31 (30.1) | | 103 | | 2.44 | 1.54 | 3.87 | 17 (28.3) | | 60 | | 1.86 | 1.01 | 3.42 |
| 29. Hypertension + Stroke | | | 111 (34.9) | | 318 | | 2.31 | 1.76 | 3.05 | 84 (43.1) | | 195 | | 2.63 | 1.90 | 3.63 |
| 30. Hypertension + Osteoporosis | | | 16 (34.0) | | 47 | | 2.24 | 1.16 | 4.34 | 155 (28.9) | | 536 | | 1.43 | 1.14 | 1.80 |
| 31. Pulmonary disease + Cancer | | | 5 (41.7) | | 12 | | 3.43 | 0.99 | 11.85 | 1 (50.0) | | 2 | | 6.57 | 0.39 | 112.08 |
| 32. Pulmonary disease + Stroke | | | 5 (83.3) | | 6 | | 18.15 | 1.95 | 169.07 | 5 (55.6) | | 9 | | 4.01 | 1.01 | 15.86 |
| (Continued to next page) | | | | | | | | | | | | | | | | |

| Supplementary Material 4. Continued |  |  | |  | |  |  | | |  | | |  | |  | |  |  | |  |  | |  | | |  | | |  |  |
| --- | --- | --- | --- | --- | --- | --- | --- | --- | --- | --- | --- | --- | --- | --- | --- | --- | --- | --- | --- | --- | --- | --- | --- | --- | --- | --- | --- | --- | --- | --- |
|  | | |  |  |  | | |  |  | |  |  | |  | |  | | |  | |  |  | |  |  | |  | | |  |
| Chronic condition combinations | Men | | | | | | | | | | | | | | Women | | | | | | | | | | | | | | |  |
|  | Depression | | | | | | | | | | | | | | Depression | | | | | | | | | | | | | | |  |
|  | Yes | | | Total | | | OR | | | 95% CI | | | | | Yes | | | Total | | | OR | | 95% CI | | | | | | |  |
|  | N (%) | | | N | | |  |  |  |  |  |  |  |  | N (%) | | | N | | |  |  |  |  |  |  |  |  |  |  |
| 33. Pulmonary disease + Osteoporosis | 2 (40.0) | | | 5 | | | 2.23 | | | 0.30 | | | 16.30 | | 8 (33.3) | | | 24 | | | 2.18 | | 0.89 | | | 5.34 | | |  |  |
| 34. Cancer + Stroke | 3 (50.0) | | | 6 | | | 4.50 | | | 0.73 | | | 27.92 | | 2 (66.7) | | | 3 | | | 3.11 | | 0.28 | | | 34.52 | | |  |  |
| 35. Cancer + Osteoporosis | 0 (0.0) | | | 1 | | | - | | | - | | | - | | 8 (38.1) | | | 21 | | | 3.22 | | 1.23 | | | 8.43 | | |  |  |
| 36. Stroke + Osteoporosis | - | | | - | | | - | | | - | | | - | | 4 (26.7) | | | 15 | | | 1.09 | | 0.32 | | | 3.74 | | |  |  |
| Three | | |  |  |  | | | |  | | |  | |  | |  | | |  | | |  | | |  | |  | | |  |
| 37. Arthritis + Diabetes + Heart disease | 7 (46.7) | | | 15 | | | 3.29 | | | 1.09 | | | 10.00 | | 13 (39.4) | | | 33 | | | 2.02 | | 0.96 | | | 4.26 | | |  |  |
| 38. Arthritis + Diabetes + Hypertension | 84 (33.6) | | | 250 | | | 2.25 | | | 1.66 | | | 3.06 | | 269 (36.8) | | | 732 | | | 1.92 | | 1.58 | | | 2.34 | | |  |  |
| 39. Arthritis + Diabetes + Pulmonary disease | 3 (33.3) | | | 9 | | | 2.12 | | | 0.50 | | | 9.12 | | 3 (33.3) | | | 9 | | | 1.44 | | 0.34 | | | 6.03 | | |  |  |
| 40. Arthritis + Diabetes + Cancer | 1 (20.0) | | | 5 | | | 0.71 | | | 0.07 | | | 7.16 | | 8 (53.3) | | | 15 | | | 4.99 | | 1.69 | | | 14.75 | | |  |  |
| 41. Arthritis + Diabetes + Stroke | 1 (25.0) | | | 4 | | | 0.99 | | | 0.09 | | | 11.12 | | 6 (54.6) | | | 11 | | | 4.97 | | 1.31 | | | 18.82 | | |  |  |
| 42. Arthritis + Diabetes + Osteoporosis | 0 (0.0) | | | 4 | | | - | | | - | | | - | | 36 (32.4) | | | 111 | | | 1.46 | | 0.94 | | | 2.26 | | |  |  |
| 43. Arthritis + Heart disease + Hypertension | 26 (28.3) | | | 92 | | | 1.76 | | | 1.08 | | | 2.88 | | 139 (42.5) | | | 327 | | | 2.34 | | 1.80 | | | 3.04 | | |  |  |
| 44. Arthritis + Heart disease + Pulmonary disease | 1 (33.3) | | | 3 | | | 0.66 | | | 0.06 | | | 7.68 | | 6 (66.7) | | | 9 | | | 4.19 | | 1.00 | | | 17.48 | | |  |  |
| 45. Arthritis + Heart disease + Cancer | 3 (42.9) | | | 7 | | | 2.65 | | | 0.55 | | | 12.68 | | - | | | - | | | - | | - | | | - | | |  |  |
| 46. Arthritis + Heart disease + Stroke | 3 (50.0) | | | 6 | | | 2.61 | | | 0.45 | | | 15.07 | | 4 (50.0) | | | 8 | | | 2.72 | | 0.63 | | | 11.76 | | |  |  |
| 47. Arthritis + Heart disease + Osteoporosis | 3 (75.0) | | | 4 | | | 8.26 | | | 0.77 | | | 88.36 | | 31 (47.7) | | | 65 | | | 2.44 | | 1.45 | | | 4.12 | | |  |  |
| 48. Arthritis + Hypertension + Pulmonary disease | 14 (37.8) | | | 37 | | | 2.29 | | | 1.11 | | | 4.69 | | 35 (39.8) | | | 88 | | | 1.94 | | 1.22 | | | 3.09 | | |  |  |
| (Continued to next page) | | | | | | | | | | | | | | | | | | | | | | | | | | | |  |  |  |

| Supplementary Material 4. Continued | | |  |  |  |  |  |  |  |  |  |  |  |  |  |  | |
| --- | --- | --- | --- | --- | --- | --- | --- | --- | --- | --- | --- | --- | --- | --- | --- | --- | --- |
|  |  |  |  |  |  |  |  |  |  |  |  |  |  |  |  |  | |
| Chronic condition combinations | | | Men | | | | | | | Women | | | | | | |  |
|  |  |  | Depression | | | | | | | Depression | | | | | | |  |
|  |  |  | Yes | | Total | | OR | 95% CI | | Yes | | Total | | OR | 95% CI | | |
|  |  |  | N (%) | | N | |  |  |  | N (%) | | N | |  |  |  |  |
| 49. Arthritis + Hypertension + Cancer | | | 3 (21.4) | | 14 | | 0.86 | 0.20 | 3.70 | 17 (36.2) | | 47 | | 2.03 | 1.08 | 3.82 | |
| 50. Arthritis + Hypertension + Stroke | | | 34 (45.3) | | 75 | | 2.90 | 1.73 | 4.84 | 78 (48.5) | | 161 | | 2.55 | 1.80 | 3.62 | |
| 51. Arthritis + Hypertension + Osteoporosis | | | 13 (31.7) | | 41 | | 1.39 | 0.67 | 2.86 | 335 (36.9) | | 908 | | 1.83 | 1.53 | 2.02 | |
| 52. Arthritis + Pulmonary disease + Cancer | | | 2 (66.7) | | 3 | | 6.88 | 0.59 | 79.95 | 0 (0.0) | | 1 | | - | - | - | |
| 53. Arthritis + Pulmonary disease + Stroke | | | 0 (0.0) | | 1 | | - | - | - | 1 (100.0) | | 1 | | - | - | - | |
| 54. Arthritis + Pulmonary disease + Osteoporosis | | | 4 (80.0) | | 5 | | 6.31 | 0.59 | 67.45 | 20 (50.0) | | 40 | | 2.80 | 1.42 | 5.53 | |
| 55. Arthritis + Cancer + Stroke | | | 0 (0.0) | | 1 | | - | - | - | - | | - | | - | - | - | |
| 56. Arthritis + Cancer + Osteoporosis | | | 1 (100.0) | | 1 | | - | - | - | 14 (60.9) | | 23 | | 7.02 | 2.85 | 17.29 | |
| 57. Arthritis + Stroke + Osteoporosis | | | 2 (100.0) | | 2 | | - | - | - | 8 (47.1) | | 17 | | 2.84 | 1.02 | 7.95 | |
| 58. Diabetes + Heart disease + Hypertension | | | 52 (28.6) | | 182 | | 2.63 | 1.84 | 3.77 | 64 (38.3) | | 167 | | 2.45 | 1.72 | 3.47 | |
| 59. Diabetes + Heart disease + Pulmonary disease | | | 5 (50.0) | | 10 | | 3.98 | 1.06 | 14.95 | - | | - | | - | - | - | |
| 60. Diabetes + Heart disease + Cancer | | | 2 (40.0) | | 5 | | 4.64 | 0.58 | 36.88 | 0 (0.0) | | 1 | | - | - | - | |
| 61. Diabetes + Heart disease + Stroke | | | 1 (14.3) | | 7 | | 0.72 | 0.08 | 6.41 | 2 (100.0) | | 2 | | - | - | - | |
| 62. Diabetes + Heart disease + Osteoporosis | | | - | | - | | - | - | - | 5 (45.5) | | 11 | | 3.27 | 0.95 | 11.31 | |
| 63. Diabetes + Hypertension + Pulmonary disease | | | 14 (26.4) | | 53 | | 1.38 | 0.70 | 2.72 | 13 (48.2) | | 27 | | 2.79 | 1.24 | 6.25 | |
| 64. Diabetes + Hypertension + Cancer | | | 15 (31.3) | | 48 | | 2.49 | 1.27 | 4.89 | 15 (53.6) | | 28 | | 4.98 | 2.20 | 11.28 | |
| 65. Diabetes + Hypertension + Stroke | | | 51 (37.5) | | 136 | | 2.76 | 1.86 | 4.11 | 33 (37.5) | | 88 | | 2.16 | 1.35 | 3.47 | |
| (Continued to next page) | | | | | | | | | | | | | | | | |  |

| Supplementary Material 4. Continued | | |  |  |  |  |  |  |  |  |  |  |  | |  |  |  |
| --- | --- | --- | --- | --- | --- | --- | --- | --- | --- | --- | --- | --- | --- | --- | --- | --- | --- |
|  |  |  |  |  |  |  |  |  |  |  |  |  |  | |  |  |  |
| Chronic condition combinations | | | Men | | | | | | | Women | | | | | | | |
|  |  |  | Depression | | | | | | | Depression | | | | | | | |
|  |  |  | Yes | | Total | | OR | 95% CI | | Yes | | Total | | OR | | 95% CI | |
|  |  |  | N (%) | | N | |  |  |  | N (%) | | N | |  |  |  |  |
| 66. Diabetes + Hypertension + Osteoporosis | | | 6 (42.9) | | 14 | | 2.43 | 0.77 | 7.63 | 51 (34.9) | | 146 | | 1.93 | | 1.32 | 2.81 |
| 67. Diabetes + Pulmonary disease + Cancer | | | 2 (100.0) | | 2 | | - | - | - | - | | - | | - | | - | - |
| 68. Diabetes + Pulmonary disease + Stroke | | | 0 (0.0) | | 1 | | - | - | - | - | | - | | - | | - | - |
| 69. Diabetes + Pulmonary disease + Osteoporosis | | | - | | - | | - | - | - | 1 (33.3) | | 3 | | 2.30 | | 0.20 | 26.56 |
| 70. Diabetes + Cancer + Stroke | | | - | | - | | - | - | - | - | | - | | - | | - | - |
| 71. Diabetes + Cancer + Osteoporosis | | | - | | - | | - | - | - | 0 (0.0) | | 4 | | - | | - | - |
| 72. Diabetes + Stroke + Osteoporosis | | | - | | - | | - | - | - | 3 (100.0) | | 3 | | - | | - | - |
| 73. Heart disease + Hypertension + Pulmonary disease | | | 6 (24.0) | | 25 | | 1.17 | 0.44 | 3.16 | 6 (35.3) | | 17 | | 1.75 | | 0.62 | 4.93 |
| 74. Heart disease + Hypertension + Cancer | | | 6 (35.3) | | 17 | | 3.51 | 1.24 | 9.95 | 4 (44.4) | | 9 | | 2.90 | | 0.74 | 11.42 |
| 75. Heart disease + Hypertension + Stroke | | | 16 (32.0) | | 50 | | 1.84 | 0.95 | 3.55 | 11 (45.8) | | 24 | | 3.09 | | 1.30 | 7.34 |
| 76. Heart disease + Hypertension + Osteoporosis | | | 3 (37.5) | | 8 | | 2.37 | 0.53 | 10.56 | 24 (28.6) | | 84 | | 1.27 | | 0.76 | 2.11 |
| 77. Heart disease + Pulmonary disease + Cancer | | | - | | - | | - | - | - | - | | - | | - | | - | - |
| 78. Heart disease + Pulmonary disease + Stroke | | | 0 (0.0) | | 1 | | - | - | - | - | | - | | - | | - | - |
| 79. Heart disease + Pulmonary disease + Osteoporosis | | | - | | - | | - | - | - | 2 (66.7) | | 3 | | 4.26 | | 0.38 | 47.36 |
| 80. Heart disease + Cancer + Stroke | | | 0 (0.0) | | 2 | | - | - | - | - | | - | | - | | - | - |
| 81. Heart disease + Cancer + Osteoporosis | | | 1 (100.0) | | 1 | | - | - | - | 1 (100.0) | | 1 | | - | | - | - |
| 82. Heart disease + Stroke + Osteoporosis | | | 1 (100.0) | | 1 | | - | - | - | 1 (100.0) | | 1 | | - | | - | - |
| (Continued to next page) | | | | | | | | | | | | | | | | | |

| Supplementary Material 4. Continued | | |  |  |  |  |  |  |  |  |  |  |  |  |  |  |
| --- | --- | --- | --- | --- | --- | --- | --- | --- | --- | --- | --- | --- | --- | --- | --- | --- |
|  |  |  |  |  |  |  |  |  |  |  |  |  |  |  |  |  |
| Chronic condition combinations | | | Men | | | | | | | Women | | | | | | |
|  |  |  | Depression | | | | | | | Depression | | | | | | |
|  |  |  | Yes | | Total | | OR | 95% CI | | Yes | | Total | | OR | 95% CI | |
|  |  |  | N (%) | | N | |  |  |  | N (%) | | N | |  |  |  |
| 83. Hypertension + Pulmonary disease + Cancer | | | 6 (85.7) | | 7 | | 23.01 | 2.38 | 222.18 | 1 (50.0) | | 2 | | 1.63 | 0.06 | 46.39 |
| 84. Hypertension + Pulmonary disease + Stroke | | | 7 (36.8) | | 19 | | 2.13 | 0.77 | 5.93 | 1 (20.0) | | 5 | | 1.23 | 0.12 | 12.85 |
| 85. Hypertension + Pulmonary disease + Osteoporosis | | | 0 (0.0) | | 1 | | - | - | - | 8 (40.0) | | 20 | | 2.10 | 0.81 | 5.44 |
| 86. Hypertension + Cancer + Stroke | | | 3 (27.3) | | 11 | | 2.04 | 0.50 | 8.39 | 2 (33.3) | | 6 | | 1.40 | 0.23 | 8.76 |
| 87. Hypertension + Cancer + Osteoporosis | | | 2 (100.0) | | 2 | | - | - | - | 5 (25.0) | | 20 | | 1.06 | 0.36 | 3.11 |
| 88. Hypertension + Stroke + Osteoporosis | | | - | | - | | - | - | - | 16 (42.1) | | 38 | | 2.73 | 1.37 | 5.43 |
| 89. Pulmonary disease + Cancer + Stroke | | | - | | - | | - | - | - | - | | - | | - | - | - |
| 90. Pulmonary disease + Cancer + Osteoporosis | | | 0 (0.0) | | 1 | | - | - | - | 2 (66.7) | | 3 | | 6.03 | 0.53 | 68.47 |
| 91. Pulmonary disease + Stroke + Osteoporosis | | | 0 (0.0) | | 1 | | - | - | - | - | | - | | - | - | - |
| 92. Cancer + Stroke + Osteoporosis | | | - | | - | | - | - | - | - | | - | | - | - | - |
| Four |  |  |  | |  | |  |  |  |  | |  | |  |  |  |
| 93. Arthritis + Diabetes + Heart disease + Hypertension | | | 13 (48.2) | | 27 | | 4.18 | 1.85 | 9.42 | 77 (45.6) | | 169 | | 2.53 | 1.80 | 3.56 |
| 94. Arthritis + Diabetes + Heart disease + Pulmonary disease | | | 1 (50.0) | | 2 | | 3.42 | 0.18 | 66.26 | 1 (50.0) | | 2 | | 6.48 | 0.40 | 105.42 |
| 95. Arthritis + Diabetes + Heart disease + Cancer | | | 2 (100.0) | | 2 | | - | - | - | - | | - | | - | - | - |
| 96. Arthritis + Diabetes + Heart disease + Stroke | | | 0 (0.0) | | 1 | | - | - | - | 1 (100.0) | | 1 | | - | - | - |
| 97. Arthritis + Diabetes + Heart disease + Osteoporosis | | | - | | - | | - | - | - | 4 (33.3) | | 12 | | 1.44 | 0.41 | 5.12 |
| 98. Arthritis + Diabetes + Hypertension + Pulmonary disease | | | 6 (50.0) | | 12 | | 4.30 | 1.28 | 14.42 | 13 (52.0) | | 25 | | 2.61 | 1.13 | 6.06 |
| (Continued to next page) | | | | | | | | | | | | | | | | |

| Supplementary Material 4. Continued | | |  |  |  |  |  |  |  |  |  |  |  |  |  |  |
| --- | --- | --- | --- | --- | --- | --- | --- | --- | --- | --- | --- | --- | --- | --- | --- | --- |
|  |  |  |  |  |  |  |  |  |  |  |  |  |  |  |  |  |
| Chronic condition combinations | | | Men | | | | | | | Women | | | | | | |
|  |  |  | Depression | | | | | | | Depression | | | | | | |
|  |  |  | Yes | | Total | | OR | 95% CI | | Yes | | Total | | OR | 95% CI | |
|  |  |  | N (%) | | N | |  |  |  | N (%) | | N | |  |  |  |
| 99. Arthritis + Diabetes + Hypertension + Cancer | | | 3 (42.9) | | 7 | | 4.37 | 0.91 | 20.89 | 12 (37.5) | | 32 | | 2.13 | 0.98 | 4.62 |
| 100. Arthritis + Diabetes + Hypertension + Stroke | | | 23 (53.5) | | 43 | | 4.51 | 2.31 | 8.82 | 50 (60.2) | | 83 | | 4.65 | 2.88 | 7.52 |
| 101. Arthritis + Diabetes + Hypertension + Osteoporosis | | | 4 (44.4) | | 9 | | 3.31 | 0.80 | 13.65 | 139 (42.5) | | 327 | | 2.25 | 1.73 | 2.91 |
| 102. Arthritis + Diabetes + Pulmonary disease + Cancer | | | - | | - | | - | - | - | - | | - | | - | - | - |
| 103. Arthritis + Diabetes + Pulmonary disease + Stroke | | | 1 (100.0) | | 1 | | - | - | - | - | | - | | - | - | - |
| 104. Arthritis + Diabetes + Pulmonary disease + Osteoporosis | | | 1 (100.0) | | 1 | | - | - | - | 1 (50.0) | | 2 | | 4.01 | 0.25 | 65.22 |
| 105. Arthritis + Diabetes + Cancer + Stroke | | | - | | - | | - | - | - | 1 (50.0) | | 2 | | 2.84 | 0.15 | 53.29 |
| 106. Arthritis + Diabetes + Cancer + Osteoporosis | | | - | | - | | - | - | - | 0 (0.0) | | 1 | | - | - | - |
| 107. Arthritis + Diabetes + Stroke + Osteoporosis | | | - | | - | | - | - | - | 5 (83.3) | | 6 | | 17.57 | 1.91 | 162.00 |
| 108. Arthritis + Heart disease + Hypertension + Pulmonary disease | | | 8 (66.7) | | 12 | | 8.31 | 2.30 | 30.08 | 14 (58.3) | | 24 | | 3.02 | 1.29 | 7.05 |
| 109. Arthritis + Heart disease + Hypertension + Cancer | | | - | | - | | - | - | - | 3 (50.0) | | 6 | | 4.67 | 0.91 | 23.89 |
| 110. Arthritis + Heart disease + Hypertension + Stroke | | | 10 (52.6) | | 19 | | 3.79 | 1.35 | 10.61 | 19 (61.3) | | 31 | | 4.78 | 2.25 | 10.19 |
| 111. Arthritis + Heart disease + Hypertension + Osteoporosis | | | 1 (16.7) | | 6 | | 0.53 | 0.06 | 5.03 | 55 (39.6) | | 139 | | 1.97 | 1.35 | 2.88 |
| 112. Arthritis + Heart disease + Pulmonary disease + Cancer | | | - | | - | | - | - | - | - | | - | | - | - | - |
| 113. Arthritis + Heart disease + Pulmonary disease + Stroke | | | - | | - | | - | - | - | - | | - | | - | - | - |
| 114. Arthritis + Heart disease + Pulmonary disease + Osteoporosis | | | - | | - | | - | - | - | 4 (50.0) | | 8 | | 1.72 | 0.41 | 7.23 |
| 115. Arthritis + Heart disease + Cancer + Stroke | | | - | | - | | - | - | - | - | | - | | - | - | - |
| (Continued to next page) | | | | | | | | | | | | | | | | |

| Supplementary Material 4. Continued | | |  |  |  |  |  |  |  |  |  |  |  |  |  |  |
| --- | --- | --- | --- | --- | --- | --- | --- | --- | --- | --- | --- | --- | --- | --- | --- | --- |
|  |  |  |  |  |  |  |  |  |  |  |  |  |  |  |  |  |
| Chronic condition combinations | | | Men | | | | | | | Women | | | | | | |
|  |  |  | Depression | | | | | | | Depression | | | | | | |
|  |  |  | Yes | | Total | | OR | 95% CI | | Yes | | Total | | OR | 95% CI | |
|  |  |  | N (%) | | N | |  |  |  | N (%) | | N | |  |  |  |
| 116. Arthritis + Heart disease + Cancer + Osteoporosis | | | - | | - | | - | - | - | 4 (100.0) | | 4 | | - | - | - |
| 117. Arthritis + Heart disease + Stroke + Osteoporosis | | | - | | - | | - | - | - | 1 (100.0) | | 1 | | - | - | - |
| 118. Arthritis + Hypertension + Pulmonary disease + Cancer | | | - | | - | | - | - | - | 3 (75.0) | | 4 | | 4.56 | 0.44 | 47.76 |
| 119. Arthritis + Hypertension + Pulmonary disease + Stroke | | | 5 (71.4) | | 7 | | 5.13 | 0.92 | 28.75 | 5 (83.3) | | 6 | | 17.14 | 1.70 | 172.39 |
| 120. Arthritis + Hypertension + Pulmonary disease + Osteoporosis | | | 1 (50.0) | | 2 | | 4.11 | 0.26 | 66.24 | 31 (50.8) | | 61 | | 2.69 | 1.56 | 4.65 |
| 121. Arthritis + Hypertension + Cancer + Stroke | | | 1 (33.3) | | 3 | | 3.84 | 0.35 | 42.65 | 3 (50.0) | | 6 | | 2.18 | 0.41 | 11.59 |
| 122. Arthritis + Hypertension + Cancer + Osteoporosis | | | 0 (0.0) | | 4 | | - | - | - | 5 (20.8) | | 24 | | 0.97 | 0.35 | 2.69 |
| 123. Arthritis + Hypertension + Stroke + Osteoporosis | | | 4 (57.1) | | 7 | | 7.68 | 1.63 | 36.25 | 52 (55.9) | | 93 | | 3.46 | 2.21 | 5.42 |
| 124. Arthritis + Pulmonary disease + Cancer + Stroke | | | - | | - | | - | - | - | - | | - | | - | - | - |
| 125. Arthritis + Pulmonary disease + Cancer + Osteoporosis | | | - | | - | | - | - | - | 1 (33.3) | | 3 | | 1.34 | 0.09 | 21.03 |
| 126. Arthritis + Pulmonary disease + Stroke + Osteoporosis | | | 1 (100.0) | | 1 | | - | - | - | 0 (0.0) | | 2 | | - | - | - |
| 127. Arthritis + Cancer + Stroke + Osteoporosis | | | - | | - | | - | - | - | - | | - | | - | - | - |
| 128. Diabetes + Heart disease + Hypertension + Pulmonary disease | | | 7 (50.0) | | 14 | | 4.69 | 1.53 | 14.42 | 5 (45.5) | | 11 | | 2.28 | 0.68 | 7.73 |
| 129. Diabetes + Heart disease + Hypertension + Cancer | | | 5 (41.7) | | 12 | | 4.09 | 1.20 | 13.96 | 0 (0.0) | | 3 | | - | - | - |
| 130. Diabetes + Heart disease + Hypertension + Stroke | | | 19 (50.0) | | 38 | | 5.62 | 2.78 | 11.38 | 12 (60.0) | | 20 | | 4.76 | 1.83 | 12.40 |
| 131. Diabetes + Heart disease + Hypertension + Osteoporosis | | | 2 (50.0) | | 4 | | 4.86 | 0.66 | 35.74 | 21 (55.3) | | 38 | | 5.09 | 2.58 | 10.06 |
| 132. Diabetes + Heart disease + Pulmonary disease + Cancer | | | - | | - | | - | - | - | - | | - | | - | - | - |
| (Continued to next page) | | | | | | | | | | | | | | | | |

| Supplementary Material 4. Continued | | |  |  |  |  |  |  |  |  |  |  |  |  |  |  |
| --- | --- | --- | --- | --- | --- | --- | --- | --- | --- | --- | --- | --- | --- | --- | --- | --- |
|  |  |  |  |  |  |  |  |  |  |  |  |  |  |  |  |  |
| Chronic condition combinations | | | Men | | | | | | | Women | | | | | | |
|  |  |  | Depression | | | | | | | Depression | | | | | | |
|  |  |  | Yes | | Total | | OR | 95% CI | | Yes | | Total | | OR | 95% CI | |
|  |  |  | N (%) | | N | |  |  |  | N (%) | | N | |  |  |  |
| 133. Diabetes + Heart disease + Pulmonary disease + Stroke | | | 0 (0.0) | | 1 | | - | - | - | - | | - | | - | - | - |
| 134. Diabetes + Heart disease + Pulmonary disease + Osteoporosis | | | - | | - | | - | - | - | - | | - | | - | - | - |
| 135. Diabetes + Heart disease + Cancer + Stroke | | | 1 (100.0) | | 1 | | - | - | - | - | | - | | - | - | - |
| 136. Diabetes + Heart disease + Cancer + Osteoporosis | | | - | | - | | - | - | - | 0 (0.0) | | 1 | | - | - | - |
| 137. Diabetes + Heart disease + Stroke + Osteoporosis | | | - | | - | | - | - | - | - | | - | | - | - | - |
| 138. Diabetes + Hypertension + Pulmonary disease + Cancer | | | 1 (50.0) | | 2 | | 5.95 | 0.37 | 96.06 | 1 (100.0) | | 1 | | - | - | - |
| 139. Diabetes + Hypertension + Pulmonary disease + Stroke | | | 4 (50.0) | | 8 | | 3.49 | 0.76 | 16.08 | 0 (0.0) | | 3 | | - | - | - |
| 140. Diabetes + Hypertension + Pulmonary disease + Osteoporosis | | | 0 (0.0) | | 2 | | - | - | - | 1 (33.3) | | 3 | | 1.57 | 0.11 | 23.34 |
| 141. Diabetes + Hypertension + Cancer + Stroke | | | 2 (28.6) | | 7 | | 2.21 | 0.41 | 11.96 | 1 (100.0) | | 1 | | - | - | - |
| 142. Diabetes + Hypertension + Cancer + Osteoporosis | | | - | | - | | - | - | - | 1 (20.0) | | 5 | | 0.87 | 0.09 | 8.13 |
| 143. Diabetes + Hypertension + Stroke + Osteoporosis | | | 1 (33.3) | | 3 | | 1.16 | 0.10 | 13.63 | 2 (22.2) | | 9 | | 1.07 | 0.21 | 5.49 |
| 144. Diabetes + Pulmonary disease + Cancer + Stroke | | | - | | - | | - | - | - | - | | - | | - | - | - |
| 145. Diabetes + Pulmonary disease + Cancer + Osteoporosis | | | 1 (50.0) | | 2 | | 3.54 | 0.21 | 61.29 | - | | - | | - | - | - |
| 146. Diabetes + Pulmonary disease + Stroke + Osteoporosis | | | - | | - | | - | - | - | - | | - | | - | - | - |
| 147. Diabetes + Cancer + Stroke + Osteoporosis | | | - | | - | | - | - | - | - | | - | | - | - | - |
| 148. Heart disease + Hypertension + Pulmonary disease + Cancer | | | - | | - | | - | - | - | - | | - | | - | - | - |
| 149. Heart disease + Hypertension + Pulmonary disease + Stroke | | | 3 (60.0) | | 5 | | 12.88 | 2.10 | 79.07 | 0 (00.0) | | 2 | | - | - | - |
| (Continued to next page) | | | | | | | | | | | | | | | | |

| Supplementary Material 4. Continued | | |  | |  | |  |  | |  |  |  |  |  |  |  | |  |  | |  | |  |
| --- | --- | --- | --- | --- | --- | --- | --- | --- | --- | --- | --- | --- | --- | --- | --- | --- | --- | --- | --- | --- | --- | --- | --- |
|  |  |  |  | |  | |  |  | |  |  |  |  |  |  |  | |  |  | |  | |  |
| Chronic condition combinations | | | Men | | | | | | | | | | Women | | | | | | | | | | |
|  |  |  | Depression | | | | | | | | | | Depression | | | | | | | | | | |
|  |  |  | Yes | Total | | OR | | | 95% CI | | | | Yes | | Total | | OR | | | 95% CI | | | |
|  |  |  | N (%) | N | |  |  |  |  |  |  |  | N (%) | | N | |  |  |  |  |  |  |  |
| 150. Heart disease + Hypertension + Pulmonary disease + Osteoporosis | | | 0 (0.0) | 2 | | - | | | - | | | - | 2 (50.0) | | 4 | | 3.18 | | | 0.40 | | 25.29 | |
| 151. Heart disease + Hypertension + Cancer + Stroke | | | 1 (100.0) | 1 | | - | | | - | | | - | 2 (66.7) | | 3 | | 4.80 | | | 0.43 | | 54.21 | |
| 152. Heart disease + Hypertension + Cancer + Osteoporosis | | | - | - | | - | | | - | | | - | 1 (33.3) | | 3 | | 1.25 | | | 0.10 | | 15.18 | |
| 153. Heart disease + Hypertension + Stroke + Osteoporosis | | | 1 (50.0) | 2 | | 1.18 | | | 0.06 | | | 24.81 | 3 (100.0) | | 3 | | - | | | - | | - | |
| 154. Heart disease + Pulmonary disease + Cancer + Stroke | | | 0 (0.0) | 1 | | - | | | - | | | - | - | | - | | - | | | - | | - | |
| 155. Heart disease + Pulmonary disease + Cancer + Osteoporosis | | | - | - | | - | | | - | | | - | - | | - | | - | | | - | | - | |
| 156. Heart disease + Pulmonary disease + Stroke + Osteoporosis | | | - | - | | - | | | - | | | - | - | | - | | - | | | - | | - | |
| 157. Heart disease + Cancer + Stroke + Osteoporosis | | | - | - | | - | | | - | | | - | - | | - | | - | | | - | | - | |
| 158. Hypertension + Pulmonary disease + Cancer + Stroke | | | 1 (50.0) | 2 | | 1.87 | | | 0.03 | | | 125.28 | - | | - | | - | | | - | | - | |
| 159. Hypertension + Pulmonary disease + Cancer + Osteoporosis | | | - | - | | - | | | - | | | - | 1 (50.0) | | 2 | | 2.11 | | | 0.11 | | 40.10 | |
| 160. Hypertension + Pulmonary disease + Stroke + Osteoporosis | | | - | - | | - | | | - | | | - | 1 (100.0) | | 1 | | - | | | - | | - | |
| 161. Hypertension + Cancer + Stroke + Osteoporosis | | | 1 (100.0) | 1 | | - | | | - | | | - | 1 (50.0) | | 2 | | 4.30 | | | 0.14 | | 137.08 | |
| 162. Pulmonary disease + Cancer + Stroke + Osteoporosis | | | - | - | | - | | | - | | | - | - | | - | | - | | | - | | - | |
| (Continued to next page) | | | | | | | | | | | | | | | | | | | | | | | |

| Supplementary Material 4. Continued | |  | |  | |  | |  |  | |  | |  | |  | |  | |  | |  |  | | |  | | |  | | | |  |  |
| --- | --- | --- | --- | --- | --- | --- | --- | --- | --- | --- | --- | --- | --- | --- | --- | --- | --- | --- | --- | --- | --- | --- | --- | --- | --- | --- | --- | --- | --- | --- | --- | --- | --- |
|  |  |  |  | |  | |  | |  |  | |  | |  | |  | |  | |  | |  | |  | | |  | | |  | | | |
| Chronic condition combinations | | Men | | | | | | | | | | | | | Women | | | | | | | | | | | | | | | |  |  |  |
|  |  | Depression | | | | | | | | | | | | | Depression | | | | | | | | | | | | | | | |  |  |  |
|  |  | Yes | | | | Total | | | OR | | 95% CI | | | | Yes | | | | Total | | | OR | | | 95% CI | | | | | | |  |  |
|  |  | N (%) | | | | N | | |  |  |  |  |  |  | N (%) | | | | N | | |  |  |  |  |  |  |  |  |  |  |  |  |
| Five |  |  |  | | | |  | | |  | |  | |  | |  | | | |  | | |  | | |  | | |  | | | |  |
| 163. Arthritis + Diabetes + Heart disease + Hypertension + Pulmonary disease | | 4 (100.0) | | | | 4 | | | - | | - | | - | | 7 (50.0) | | | | 14 | | | 2.27 | | | 0.75 | | | 6.91 | | | |  |  |
| 164. Arthritis + Diabetes + Heart disease + Hypertension + Cancer | | 1 (50.0) | | | | 2 | | | 5.96 | | 0.24 | | 148.22 | | 3 (100.0) | | | | 3 | | | - | | | - | | | - | | | |  |  |
| 165. Arthritis + Diabetes + Heart disease + Hypertension + Stroke | | 5 (55.6) | | | | 9 | | | 4.15 | | 1.00 | | 17.34 | | 12 (75.0) | | | | 16 | | | 6.45 | | | 1.95 | | | 21.40 | | | |  |  |
| 166. Arthritis + Diabetes + Heart disease + Hypertension + Osteoporosis | | 5 (83.3) | | | | 6 | | | 13.69 | | 1.48 | | 126.69 | | 38 (51.4) | | | | 74 | | | 2.85 | | | 1.73 | | | 4.68 | | | |  |  |
| 167. Arthritis + Diabetes + Heart disease + Pulmonary disease + Cancer | | - | | | | - | | | - | | - | | - | | - | | | | - | | | - | | | - | | | - | | | |  |  |
| 168. Arthritis + Diabetes + Heart disease + Pulmonary disease + Stroke | | - | | | | - | | | - | | - | | - | | 0 (0.0) | | | | 1 | | | - | | | - | | | - | | | |  |  |
| 169. Arthritis + Diabetes + Heart disease + Pulmonary disease + Osteoporosis | | - | | | | - | | | - | | - | | - | | 0 (0.0) | | | | 1 | | | - | | | - | | | - | | | |  |  |
| 170. Arthritis + Diabetes + Heart disease + Cancer + Stroke | | 0 (0.0) | | | | 1 | | | - | | - | | - | | - | | | | - | | | - | | | - | | | - | | | |  |  |
| 171. Arthritis + Diabetes + Heart disease + Cancer + Osteoporosis | | - | | | | - | | | - | | - | | - | | 0 (0.0) | | | | 2 | | | - | | | - | | | - | | | |  |  |
| 172. Arthritis + Diabetes + Heart disease + Stroke + Osteoporosis | | - | | | | - | | | - | | - | | - | | 1 (100.0) | | | | 1 | | | - | | | - | | | - | | | |  |  |
| 173. Arthritis + Diabetes + Hypertension + Pulmonary disease + Cancer | | - | | | | - | | | - | | - | | - | | 0 (0.0) | | | | 1 | | | - | | | - | | | - | | | |  |  |
| 174. Arthritis + Diabetes + Hypertension + Pulmonary disease + Stroke | | 1 (25.0) | | | | 4 | | | 1.13 | | 0.11 | | 11.53 | | 3 (100.0) | | | | 3 | | | - | | | - | | | - | | | |  |  |
| (Continued to next page) | | | | | | | | | | | | | | | | | | | | | | | | | | | | | | |  |  |  |

| Supplementary Material 4. Continued | | |  |  |  |  |  |  |  |  |  |  |  | |  | |  | |  | |
| --- | --- | --- | --- | --- | --- | --- | --- | --- | --- | --- | --- | --- | --- | --- | --- | --- | --- | --- | --- | --- |
|  |  |  |  |  |  |  |  |  |  |  |  |  |  | |  | |  | |  | |
| Chronic condition combinations | | | Men | | | | | | | Women | | | | | | | | | |  |
|  |  |  | Depression | | | | | | | Depression | | | | | | | | | |  |
|  |  |  | Yes | | Total | | OR | 95% CI | | Yes | | Total | | OR | | 95% CI | | | |  |
|  |  |  | N (%) | | N | |  |  |  | N (%) | | N | |  |  |  |  |  |  |  |
| 175. Arthritis + Diabetes + Hypertension + Pulmonary disease + Osteoporosis | | | 0 (0.0) | | 1 | | - | - | - | 15 (68.2) | | 22 | | 4.21 | | 1.63 | | 10.87 | |  |
| 176. Arthritis + Diabetes + Hypertension + Cancer + Stroke | | | 1 (100.0) | | 1 | | - | - | - | 1 (100.0) | | 1 | | - | | - | | - | |  |
| 177. Arthritis + Diabetes + Hypertension + Cancer + Osteoporosis | | | 1 (100.0) | | 1 | | - | - | - | 7 (53.9) | | 13 | | 2.67 | | 0.83 | | 8.60 | |  |
| 178. Arthritis + Diabetes + Hypertension + Stroke + Osteoporosis | | | 3 (75.0) | | 4 | | 15.61 | 1.42 | 171.92 | 16 (50.0) | | 32 | | 2.51 | | 1.20 | | 5.26 | |  |
| 179. Arthritis + Diabetes + Pulmonary disease + Cancer + Stroke | | | - | | - | | - | - | - | - | | - | | - | | - | | - | |  |
| 180. Arthritis + Diabetes + Pulmonary disease + Cancer + Osteoporosis | | | - | | - | | - | - | - | - | | - | | - | | - | | - | |  |
| 181. Arthritis + Diabetes + Pulmonary disease + Stroke + Osteoporosis | | | - | | - | | - | - | - | - | | - | | - | | - | | - | |  |
| 182. Arthritis + Diabetes + Cancer + Stroke + Osteoporosis | | | - | | - | | - | - | - | - | | - | | - | | - | | - | |  |
| 183. Arthritis + Heart disease + Hypertension + Pulmonary disease + Cancer | | | - | | - | | - | - | - | - | | - | | - | | - | | - | |  |
| 184. Arthritis + Heart disease + Hypertension + Pulmonary disease + Stroke | | | 1 (50.0) | | 2 | | 1.87 | 0.08 | 44.12 | 0 (0.0) | | 1 | | - | | - | | - | |  |
| 185. Arthritis + Heart disease + Hypertension + Pulmonary disease + Osteoporosis | | | 2 (50.0) | | 4 | | 6.48 | 0.86 | 48.84 | 7 (53.9) | | 13 | | 2.75 | | 0.90 | | 8.38 | |  |
| 186. Arthritis + Heart disease + Hypertension + Cancer + Stroke | | | 1 (50.0) | | 2 | | 6.30 | 0.35 | 113.31 | 1 (100.0) | | 1 | | - | | - | | - | |  |
| 187. Arthritis + Heart disease + Hypertension + Cancer + Osteoporosis | | | 1 (100.0) | | 1 | | - | - | - | 3 (75.0) | | 4 | | 7.42 | | 0.76 | | 72.64 | |  |
| (Continued to next page) | | | | | | | | | | | | | | | | | | | |  |

| Supplementary Material 4. Continued | | |  |  |  |  |  |  |  |  |  |  |  |  |  |  |
| --- | --- | --- | --- | --- | --- | --- | --- | --- | --- | --- | --- | --- | --- | --- | --- | --- |
|  |  |  |  |  |  |  |  |  |  |  |  |  |  |  |  |  |
| Chronic condition combinations | | | Men | | | | | | | Women | | | | | | |
|  |  |  | Depression | | | | | | | Depression | | | | | | |
|  |  |  | Yes | | Total | | OR | 95% CI | | Yes | | Total | | OR | 95% CI | |
|  |  |  | N (%) | | N | |  |  |  | N (%) | | N | |  |  |  |
| 188. Arthritis + Heart disease + Hypertension + Stroke + Osteoporosis | | | 1 (100.0) | | 1 | | - | - | - | 11 (61.1) | | 18 | | 3.51 | 1.27 | 9.68 |
| 189. Arthritis + Heart disease + Pulmonary disease + Cancer + Stroke | | | - | | - | | - | - | - | - | | - | | - | - | - |
| 190. Arthritis + Heart disease + Pulmonary disease + Cancer + Osteoporosis | | | - | | - | | - | - | - | - | | - | | - | - | - |
| 191. Arthritis + Heart disease + Pulmonary disease + Stroke + Osteoporosis | | | - | | - | | - | - | - | 3 (75.0) | | 4 | | 10.07 | 1.02 | 99.19 |
| 192. Arthritis + Heart disease + Cancer + Stroke + Osteoporosis | | | 1 (100.0) | | 1 | | - | - | - | - | | - | | - | - | - |
| 193. Arthritis + Hypertension + Pulmonary disease + Cancer + Stroke | | | - | | - | | - | - | - | 0 (0.0) | | 1 | | - | - | - |
| 194. Arthritis + Hypertension + Pulmonary disease + Cancer + Osteoporosis | | | - | | - | | - | - | - | 1 (100.0) | | 1 | | - | - | - |
| 195. Arthritis + Hypertension + Pulmonary disease + Stroke + Osteoporosis | | | - | | - | | - | - | - | 5 (62.5) | | 8 | | 3.94 | 0.86 | 18.07 |
| 196. Arthritis + Hypertension + Cancer + Stroke + Osteoporosis | | | - | | - | | - | - | - | 1 (100.0) | | 1 | | - | - | - |
| 197. Arthritis + Pulmonary disease + Cancer + Stroke + Osteoporosis | | | - | | - | | - | - | - | - | | - | | - | - | - |
| 198. Diabetes + Heart disease + Hypertension + Pulmonary disease + Cancer | | | - | | - | | - | - | - | 0 (0.0) | | 1 | | - | - | - |
| 199. Diabetes + Heart disease + Hypertension + Pulmonary disease + Stroke | | | 3 (60.0) | | 5 | | 7.91 | 1.11 | 56.56 | - | | - | | - | - | - |
| 200. Diabetes + Heart disease + Hypertension + Pulmonary disease + Osteoporosis | | | 0 (0.0) | | 2 | | - | - | - | 2 (50.0) | | 4 | | 6.95 | 0.94 | 51.38 |
| (Continued to next page) | | | | | | | | | | | | | | | | |

| Supplementary Material 4. Continued | | |  |  |  |  |  |  |  |  |  |  |  |  |  |  |
| --- | --- | --- | --- | --- | --- | --- | --- | --- | --- | --- | --- | --- | --- | --- | --- | --- |
|  |  |  |  |  |  |  |  |  |  |  |  |  |  |  |  |  |
| Chronic condition combinations | | | Men | | | | | | | Women | | | | | | |
|  |  |  | Depression | | | | | | | Depression | | | | | | |
|  |  |  | Yes | | Total | | OR | 95% CI | | Yes | | Total | | OR | 95% CI | |
|  |  |  | N (%) | | N | |  |  |  | N (%) | | N | |  |  |  |
| 201. Diabetes + Heart disease + Hypertension + Cancer + Stroke | | | - | | - | | - | - | - | 1 (100.0) | | 1 | | - | - | - |
| 202. Diabetes + Heart disease + Hypertension + Cancer + Osteoporosis | | | - | | - | | - | - | - | 0 (0.0) | | 1 | | - | - | - |
| 203. Diabetes + Heart disease + Hypertension + Stroke + Osteoporosis | | | 1 (100.0) | | 1 | | - | - | - | 1 (100.0) | | 1 | | - | - | - |
| 204. Diabetes + Heart disease + Pulmonary disease + Cancer + Stroke | | | - | | - | | - | - | - | - | | - | | - | - | - |
| 205. Diabetes + Heart disease + Pulmonary disease + Cancer + Osteoporosis | | | - | | - | | - | - | - | - | | - | | - | - | - |
| 206. Diabetes + Heart disease + Pulmonary disease + Stroke + Osteoporosis | | | - | | - | | - | - | - | - | | - | | - | - | - |
| 207. Diabetes + Heart disease + Cancer + Stroke + Osteoporosis | | | - | | - | | - | - | - | - | | - | | - | - | - |
| 208. Diabetes + Hypertension + Pulmonary disease + Cancer + Stroke | | | 0 (0.0) | | 2 | | - | - | - | - | | - | | - | - | - |
| 209. Diabetes + Hypertension + Pulmonary disease + Cancer + Osteoporosis | | | - | | - | | - | - | - | - | | - | | - | - | - |
| 210. Diabetes + Hypertension + Pulmonary disease + Stroke + Osteoporosis | | | - | | - | | - | - | - | 1 (100.0) | | 1 | | - | - | - |
| 211. Diabetes + Hypertension + Cancer + Stroke + Osteoporosis | | | - | | - | | - | - | - | - | | - | | - | - | - |
| 212. Diabetes + Pulmonary disease + Cancer + Stroke + Osteoporosis | | | - | | - | | - | - | - | - | | - | | - | - | - |
| 213. Heart disease + Hypertension + Pulmonary disease + Cancer + Stroke | | | - | | - | | - | - | - | - | | - | | - | - | - |
| (Continued to next page) | | | | | | | | | | | | | | | | |

| Supplementary Material 4. Continued | | |  |  |  |  |  |  |  |  |  |  |  |  |  |  |
| --- | --- | --- | --- | --- | --- | --- | --- | --- | --- | --- | --- | --- | --- | --- | --- | --- |
|  |  |  |  |  |  |  |  |  |  |  |  |  |  |  |  |  |
| Chronic condition combinations | | | Men | | | | | | | Women | | | | | | |
|  |  |  | Depression | | | | | | | Depression | | | | | | |
|  |  |  | Yes | | Total | | OR | 95% CI | | Yes | | Total | | OR | 95% CI | |
|  |  |  | N (%) | | N | |  |  |  | N (%) | | N | |  |  |  |
| 214. Heart disease + Hypertension + Pulmonary disease + Cancer + Osteoporosis | | | - | | - | | - | - | - | - | | - | | - | - | - |
| 215. Heart disease + Hypertension + Pulmonary disease + Stroke + Osteoporosis | | | - | | - | | - | - | - | 1 (100.0) | | 1 | | - | - | - |
| 216. Heart disease + Hypertension + Cancer + Stroke + Osteoporosis | | | - | | - | | - | - | - | - | | - | | - | - | - |
| 217. Heart disease + Pulmonary disease + Cancer + Stroke + Osteoporosis | | | - | | - | | - | - | - | - | | - | | - | - | - |
| 218. Hypertension + Pulmonary disease + Cancer + Stroke + Osteoporosis | | | - | | - | | - | - | - | - | | - | | - | - | - |
| Six |  |  |  | |  | |  |  |  |  | |  | |  |  |  |
| 219. Arthritis + Diabetes + Heart disease + Hypertension + Pulmonary disease + Cancer | | | - | | - | | - | - | - | - | | - | | - | - | - |
| 220. Arthritis + Diabetes + Heart disease + Hypertension + Pulmonary disease + Stroke | | | - | | - | | - | - | - | - | | - | | - | - | - |
| 221. Arthritis + Diabetes + Heart disease + Hypertension + Pulmonary disease + Osteoporosis | | | 0 (0.0) | | 1 | | - | - | - | 8 (88.9) | | 9 | | 13.06 | 1.56 | 109.15 |
| 222. Arthritis + Diabetes + Heart disease + Hypertension + Cancer + Stroke | | | - | | - | | - | - | - | - | | - | | - | - | - |
| 223. Arthritis + Diabetes + Heart disease + Hypertension + Cancer + Osteoporosis | | | - | | - | | - | - | - | 0 (0.0) | | 2 | | - | - | - |
| 224. Arthritis + Diabetes + Heart disease + Hypertension + Stroke + Osteoporosis | | | 0 (0.0) | | 2 | | - | - | - | 10 (58.8) | | 17 | | 3.16 | 1.12 | 8.94 |
| 225. Arthritis + Diabetes + Heart disease + Pulmonary disease + Cancer + Stroke | | | - | | - | | - | - | - | - | | - | | - | - | - |
| (Continued to next page) | | | | | | | | | | | | | | | | |

| Supplementary Material 4. Continued | | |  |  |  |  |  |  |  |  |  |  |  |  |  |  |
| --- | --- | --- | --- | --- | --- | --- | --- | --- | --- | --- | --- | --- | --- | --- | --- | --- |
|  |  |  |  |  |  |  |  |  |  |  |  |  |  |  |  |  |
| Chronic condition combinations | | | Men | | | | | | | Women | | | | | | |
|  |  |  | Depression | | | | | | | Depression | | | | | | |
|  |  |  | Yes | | Total | | OR | 95% CI | | Yes | | Total | | OR | 95% CI | |
|  |  |  | N (%) | | N | |  |  |  | N (%) | | N | |  |  |  |
| 226. Arthritis + Diabetes + Heart disease + Pulmonary disease + Cancer + Osteoporosis | | | - | | - | | - | - | - | - | | - | | - | - | - |
| 227. Arthritis + Diabetes + Heart disease + Pulmonary disease + Stroke + Osteoporosis | | | - | | - | | - | - | - | - | | - | | - | - | - |
| 228. Arthritis + Diabetes + Heart disease + Cancer + Stroke + Osteoporosis | | | - | | - | | - | - | - | - | | - | | - | - | - |
| 229. Arthritis + Diabetes + Hypertension + Pulmonary disease + Cancer + Stroke | | | - | | - | | - | - | - | - | | - | | - | - | - |
| 230. Arthritis + Diabetes + Hypertension + Pulmonary disease + Cancer + Osteoporosis | | | - | | - | | - | - | - | 1 (100.0) | | 1 | | - | - | - |
| 231. Arthritis + Diabetes + Hypertension + Pulmonary disease + Stroke + Osteoporosis | | | - | | - | | - | - | - | 1 (50.0) | | 2 | | 2.67 | 0.17 | 42.89 |
| 232. Arthritis + Diabetes + Hypertension + Cancer + Stroke + Osteoporosis | | | - | | - | | - | - | - | - | | - | | - | - | - |
| 233. Arthritis + Diabetes + Pulmonary disease + Cancer + Stroke + Osteoporosis | | | - | | - | | - | - | - | - | | - | | - | - | - |
| 234. Arthritis + Heart disease + Hypertension + Pulmonary disease + Cancer + Stroke | | | - | | - | | - | - | - | - | | - | | - | - | - |
| 235. Arthritis + Heart disease + Hypertension + Pulmonary disease + Cancer + Osteoporosis | | | - | | - | | - | - | - | - | | - | | - | - | - |
| 236. Arthritis + Heart disease + Hypertension + Pulmonary disease + Stroke + Osteoporosis | | | - | | - | | - | - | - | 1 (50.0) | | 2 | | 5.24 | 0.28 | 97.23 |
| 237. Arthritis + Heart disease + Hypertension + Cancer + Stroke + Osteoporosis | | | - | | - | | - | - | - | - | | - | | - | - | - |
| (Continued to next page) | | | | | | | | | | | | | | | | |

| Supplementary Material 4. Continued | |  | | |  | |  | |  | |  | |  | |  | |  | | |  | |  | | |  | |  | | |  | |  | | |  |
| --- | --- | --- | --- | --- | --- | --- | --- | --- | --- | --- | --- | --- | --- | --- | --- | --- | --- | --- | --- | --- | --- | --- | --- | --- | --- | --- | --- | --- | --- | --- | --- | --- | --- | --- | --- |
|  |  | |  |  | |  | |  | |  | |  | |  | |  | | |  | |  | | |  | |  | | |  | |  | |  | | |
| Chronic condition combinations | | Men | | | | | | | | | | | | | | | Women | | | | | | | | | | | | | | | | |  |  |
|  |  | Depression | | | | | | | | | | | | | | | Depression | | | | | | | | | | | | | | | | |  |  |
|  |  | Yes | | | | | Total | | | | OR | | 95% CI | | | | | Yes | | | | | Total | | | | | OR | | 95% CI | | | | |  |
|  |  | N (%) | | | | | N | | | |  |  |  |  |  |  |  | N (%) | | | | | N | | | | |  |  |  |  |  |  |  |  |
| 238. Arthritis + Heart disease + Pulmonary disease + Cancer + Stroke + Osteoporosis | | - | | | | | - | | | | - | | - | | - | | - | | | | | - | | | | | - | | | - | | - | | |  |
| 239. Arthritis + Hypertension + Pulmonary disease + Cancer + Stroke + Osteoporosis | | - | | | | | - | | | | - | | - | | - | | - | | | | | - | | | | | - | | | - | | - | | |  |
| 240. Diabetes + Heart disease + Hypertension + Pulmonary disease + Cancer + Stroke | | 0 (0.0) | | | | | 1 | | | | - | | - | | - | | - | | | | | - | | | | | - | | | - | | - | | |  |
| 241. Diabetes + Heart disease + Hypertension + Pulmonary disease + Cancer + Osteoporosis | | - | | | | | - | | | | - | | - | | - | | - | | | | | - | | | | | - | | | - | | - | | |  |
| 242. Diabetes + Heart disease + Hypertension + Pulmonary disease + Stroke + Osteoporosis | | 0 (0.0) | | | | | 1 | | | | - | | - | | - | | - | | | | | - | | | | | - | | | - | | - | | |  |
| 243. Diabetes + Heart disease + Hypertension + Cancer + Stroke + Osteoporosis | | - | | | | | - | | | | - | | - | | - | | - | | | | | - | | | | | - | | | - | | - | | |  |
| 244. Diabetes + Heart disease + Pulmonary disease + Cancer + Stroke + Osteoporosis | | - | | | | | - | | | | - | | - | | - | | - | | | | | - | | | | | - | | | - | | - | | |  |
| 245. Diabetes + Hypertension + Pulmonary disease + Cancer + Stroke + Osteoporosis | | - | | | | | - | | | | - | | - | | - | | - | | | | | - | | | | | - | | | - | | - | | |  |
| 246. Heart disease + Hypertension + Pulmonary disease + Cancer + Stroke + Osteoporosis | | - | | | | | - | | | | - | | - | | - | | - | | | | | - | | | | | - | | | - | | - | | |  |
| Seven |  | |  |  | | | |  | | | |  | |  | |  | | |  | | | | |  | | | | |  | |  | |  | | |
| 247. Arthritis + Diabetes + Heart disease + Hypertension + Pulmonary disease + Cancer + Stroke | | - | | | | | - | | | | - | | - | | - | | - | | | | | - | | | | | - | | | - | | - | | |  |
| 248. Arthritis + Diabetes + Heart disease + Hypertension + Pulmonary disease + Cancer + Osteoporosis | | - | | | | | - | | | | - | | - | | - | | 0 (0.0) | | | | | 1 | | | | | - | | | - | | - | | |  |
| (Continued to next page) | | | | | | | | | | | | | | | | | | | | | | | | | | | | | | | | | |  |  |

| Supplementary Material 4. Continued |  | | | |  |  | |  | | | |  | | | |  | | | |  | | | |  | | | |  | |  | | | |  | |  | | | | |  | | | |  | | | | |  |  |
| --- | --- | --- | --- | --- | --- | --- | --- | --- | --- | --- | --- | --- | --- | --- | --- | --- | --- | --- | --- | --- | --- | --- | --- | --- | --- | --- | --- | --- | --- | --- | --- | --- | --- | --- | --- | --- | --- | --- | --- | --- | --- | --- | --- | --- | --- | --- | --- | --- | --- | --- | --- |
|  | |  |  | | | |  | |  | |  | |  | | | |  | | | | |  | | |  | | | |  | |  | | | |  | | |  | | | |  | | | |  | | |  | | |
| Chronic condition combinations | Men | | | | | | | | | | | | | | | | | | | | Women | | | | | | | | | | | | | | | | | | | | | | | | | | |  |  |  |  |
|  | Depression | | | | | | | | | | | | | | | | | | | | Depression | | | | | | | | | | | | | | | | | | | | | | | | | | |  |  |  |  |
|  | Yes | | | Total | | | | | | OR | | | | | 95% CI | | | | | | | Yes | | | | | Total | | | | | | OR | | | | | | 95% CI | | | | | | | |  |  |  |  |  |
|  | N (%) | | | N | | | | | |  |  |  |  |  |  |  |  |  |  |  |  | N (%) | | | | | N | | | | | |  |  |  |  |  |  |  |  |  |  |  |  |  |  |  |  |  |  |  |
| 249. Arthritis + Diabetes + Heart disease + Hypertension + Pulmonary disease + Stroke + Osteoporosis | - | | | - | | | | | | - | | | | | - | | | - | | | | 2 (66.7) | | | | | 3 | | | | | | | 2.04 | | | | | | 0.15 | | | | 28.61 | | | |  |  |  |  |
| 250. Arthritis + Diabetes + Heart disease + Hypertension + Cancer + Stroke + Osteoporosis | - | | | - | | | | | | - | | | | | - | | | - | | | | - | | | | | - | | | | | | | - | | | | | | - | | | | - | | | |  |  |  |  |
| 251. Arthritis + Diabetes + Heart disease + Pulmonary disease + Cancer + Stroke + Osteoporosis | - | | | - | | | | | | - | | | | | - | | | - | | | | - | | | | | - | | | | | | | - | | | | | | - | | | | - | | | |  |  |  |  |
| 252. Arthritis + Diabetes + Hypertension + Pulmonary disease + Cancer + Stroke + Osteoporosis | - | | | - | | | | | | - | | | | | - | | | - | | | | - | | | | | - | | | | | | | - | | | | | | - | | | | - | | | |  |  |  |  |
| 253. Arthritis + Heart disease + Hypertension + Pulmonary disease + Cancer + Stroke + Osteoporosis | - | | | - | | | | | | - | | | | | - | | | - | | | | - | | | | | - | | | | | | | - | | | | | | - | | | | - | | | |  |  |  |  |
| 254. Diabetes + Heart disease + Hypertension + Pulmonary disease + Cancer + Stroke + Osteoporosis | - | | | - | | | | | | - | | | | | - | | | - | | | | - | | | | | - | | | | | | | - | | | | | | - | | | | - | | | |  |  |  |  |
| Eight | |  |  | | | |  |  | | | | | |  | | | | |  | | | |  | | |  | | | | | |  | | | | |  | | | | | |  | | | | |  | | |  |
| 255. Arthritis + Diabetes + Heart disease + Hypertension + Pulmonary disease + Cancer + Stroke + Osteoporosis | - | | | - | | | | | | - | | | | | - | | | - | | | | - | | | | | - | | | | | | | - | | | | | | - | | | | - | | | |  |  |  |  |
| Adjusted for age, marital status, living arrangement, education, type of insurance, current smoking, lack of exercise, high-risk alcohol drinking, restriction on activities of daily living, frequency of contact with people, other chronic conditions, and year. | | | | | | | | | | | | | | | | | | | | | | | | | | | | | | | | | | | | | | | | | | | | | | | |  |  |  |  |
|  |  |  |  |  |  |  |  |  |  |  |  |  |  |  |  |  |  |  |  |  |  |  |  |  |  |  |  |  |  |  |  |  |  |  |  |  |  |  |  |  |  |  |  |  |  |  |  |  |  |  |  |
